# Tracking the effect of roasting and fermentation on the metabolites of licorice root (*Glycyrrhiza glabra* L.) using UPLC-MS analysis combined with multivariate statistical analysis

**DOI:** 10.1186/s12906-023-04239-7

**Published:** 2023-11-20

**Authors:** Sarah S. Takla, Eman Shawky, Yasmin A. Mahgoub, Reham S. Darwish

**Affiliations:** Department of Pharmacognosy, Faculty of Pharmacy, Alexandria University, Alkhartoom square, Egypt, Alexandria 21521 Egypt

**Keywords:** Liquorice roots, Metabolomics, Roasting, Fermentation, UPLC/MS/MS, Multivariate analysis

## Abstract

**Background:**

Roasting, honey-roasting and fermentation are the most common pre-processing procedures of licorice roots. They were shown to noticeably change the composition of extracts. In this work, the common alterations in licorice secondary metabolites by processing were interpreted. Comprehensive metabolic profiling of different studied samples was undergone.

**Methods:**

UPLC-QqQ-MS/MS analysis coupled to various chemometric analysis models was implemented to unravel the effect of different pre-processing procedures on the chemical profile of licorice samples.

**Results:**

UPLC-QqQ-MS/MS analysis designated 133 chromatographic peaks with saponins, flavonoids, chalcones and pterocarpans being the most abundant groups. Triterpene saponins dominated the secondary metabolites in the aqueous extracts, with fermented samples showing the highest relative amounts. Meanwhile the ethanol extracts showed significant amounts of chalcones. Melanoidins were only detected in roasted and honey roasted samples. Multivariate models indicated that roasting of samples induced a greater effect on the polar metabolites rather than nonpolar ones. Variable of importance (VIP) plot indicated that glycyrrhizin and its hydrolysis product glycyrrhetinic acid, trihdroxychalcone diglycoside, glabrone and glabridin are the main chemical features responsible for the discrimination of samples.

**Conclusion:**

Coupling UPLC-MS/MS to multivariate analysis was a successful tool that unveiled the significant effect of different pre-processing methods on the chemical profile of processed and unprocessed licorice samples. Moreover, such coupling unraveled the discriminatory chemical compounds among tested samples that can be employed as markers for the processing procedure of licorice.

**Supplementary Information:**

The online version contains supplementary material available at 10.1186/s12906-023-04239-7.

## Background

*Glycyrrhiza* genus, belonging to family Fabaceae, encompasses more than 30 species, broadly scattered worldwide [1]. The genus is one of the most comprehensively researched medicinal plants of the globe [1] and is one of the most frequently prescribed natural medicine in China [2, 3]. Medicinal uses of licorice are recorded in ancient texts such as the Assyrian Herbal (2000 BC) and Ebers Papyrus (1600 BC) [1]. The rhizomes and roots are the most valuable parts used in medicinal and pharmaceutical activities and in the production of food supplements and functional foods [4].

The most widely distributed species is *Glycyrrhiza glabra* [1]. It is a very well-known traditional medicine and natural sweetener. Owing to its broad range of outstanding pharmacological effects, it is nowadays globally used in food, beverages, nutraceuticals, and herbal industry [5].

More than 400 compounds have been discovered in licorice, comprising triterpene saponins, flavonoids, pterocarpan, coumarins, phenolics, and others [6]. The biologically active compounds of licorice are being employed as plant-based remedies for numerous disease conditions due to their antioxidant, neuroprotective, anti-inflammatory, antiviral, and anti-carcinogenic assets, in addition to their ability to treat glucose intolerance and improve insulin sensitivity. Many of those pharmacological properties in addition to other medicinal benefits of licorice are supported by numerous clinical studies [7].

Roasting, honey-roasting and fermentation are the most common pre-processing procedures of herbal preparations. Roasting and honey-roasting of herbal drugs roots to primeval periods in China [8]. The Japanese Pharmacopoeia describes prepared *Glycyrrhiza* as roasted licorice, and the Chinese Pharmacopoeia describes Glycyrrhizae Radix et Rhizoma Preparata Cum Melle as honey-roasted licorice [8]. Roasting is a dry heat processing treatment that is known improve the flavor of a licorice extract [9]. Moreover, honey has natural deep eutectic solvent (NADES) characteristics [10]. Consequently, the roasting and honey roasting procedures could have a positive effect on the extraction process of licorice and increase the antioxidant activity. For several decades, fermented natural beverages have been a component of regular food consumption for many people [11]. Fermentation portrays a significant role in the production of many enzymes. The health benefits of fermented beverages and powerful functional food spawns a prospective for using fermentation as a pre-processing step in natural product extraction.

It was shown by several research groups that different pre-processing procedures can noticeably increase [12] or decrease [13] the amount, in addition to change the composition of herbal extract ingredients [8, 14] which was translated by variations in HPLC chromatograms (fingerprinting analysis) [8, 13–17].

In view of the above-mentioned points, this work aims at tracking and interpretation of the common alterations in the secondary metabolites comprised in raw licorice by pre-processing (roasting, honey-roasting, and aqueous fermentation) and whether those chemical variations can be employed as markers for scrutinizing processed and unprocessed samples.

## Methods

### Plant collection

Licorice (*Glycyrrhiza glabra*) was purchased from the local market, Alexandria, Egypt in January 2022. Based on macroscopical and microscopical characteristics, specimens were kindly authenticated by Prof. Sania Ahmed, Faculty of Science, Alexandria University. A voucher (GG 22) specimen was deposited in the Department of Pharmacognosy, Faculty of Pharmacy, Alexandria University.

### Sample preparation

All the purchased samples were ground using an electric grinder. Based on the pre-processing method, samples were divided into 4 categories: raw, roasted, honey-roasted and fermented.

The total number of samples was 21, three of which were fermented samples. Raw, roasted and honey roasted samples were studied in two groups, The first group included 9 samples (3 samples for each category) that used to prepare ethanol extracts and the second group also included 9 samples (3 samples for each category) that used to prepare aqueous extracts Table S1.

Roasted licorice samples were prepared by drying samples in the oven at 180^◦^C till constant weight (1 h) [18]. Honey-roasted samples were prepared by mixing 50 g of the powdered licorice with 12.5 g honey dissolved in 25 mL water followed by sample roasting in oven at 180^◦^C for 1 h. [19]. Afterwards, ethanolic extraction of raw, roasted and honey-roasted licorice was undergone by maceration of each sample (50 g) separately in 95% ethanol followed by concentration to dryness under reduced pressure. On the other hand, aqueous extracts of raw, roasted and honey-roasted licorice were prepared by maceration of each sample separately in distilled water followed by their filtration and lyophilization. Finally, fermentation of licorice was done by mixing 50 g sample with 50 mL water at 20–25 ^◦^C and this mixture was subjected to milling using a mortar and a pestle to get a paste. The paste is then kept in darkness for 2 days followed by soaking in water overnight. The resultant juice (fermented licorice sample (*Erk-sous* beverage)) was obtained through filtration [20]. The resulted filtrates were then lyophilized.

### Chemical profiling of processed and unprocessed licorice extracts using UPLC-MS/MS

#### Preparation of extracts sample for UPLC-MS analysis

The dry extracts were prepared at a concentration of 1 mg/mL using HPLC-grade methanol (Merck, Germany), then filtered using membrane disc filter (0.2 μm). Moreover, samples degassing was performed before injection via sonication. The injection volume of each sample was 10 µL, introduced to the chromatographic column in the full loop mode. The analysis of each sample was repeated five times.

#### Conditions of the UPLC experiment

The metabolic profiles of *G. glabra* extracts were assessed using an UPLC XEVO TQD triple quadruple instrument (Waters Corporation, Milford, MA01757 U.S.A).

The chromatographic system consisted of: a Waters Acquity QSM pump, a LC-2040 autosampler, degasser and Waters Acquity CM detector. Waters Acquity UPLC BEH C18 column 50 mm (L), 2.1 mm (ID) and 1.7 μm (PS). The flow rate was 0.2 mL/ min and the temperature was adjusted at 30 °C.

The mobile phase that was used for analyses consisted of two phases; A and B. Ultrapure water + 0.1%(v/v) formic acid (Phase A), and methanol + 0.1% (v/v) formic acid (Phase B). These mobile phase components were selected after several trials to obtain the best possible separation and resolution [21]. Gradient elution order was as following: 0.0–2.0 min, 10% eluent B; 2.0–5.0 min, 30% eluent B; 5.0–15.0 min, 70% eluent B; 15.0-22.0 min, 90% eluent B; 22.0–25.0 min, 90% eluent B; 26.0 min, 100% eluent B; 26.0–29.0 min, 100% eluent B; 30.0–40 min, 10% eluent B. The post time was set for 4 min for column re-equilibration using methanol.

#### Conditions of ESI-MS and metabolites annotation

Negative and positive ionization modes were used for samples analysis, the mass analyzer was the triple quadrupole (TQD) mass spectrometer, accompanied by electrospray ionization (ESI) source.

To get a comprehensive picture of the metabolite profiles of the test extracts, the electrospray ionization source (ESI) was set to be in the negative and positive ion modes. The mass analyzer used was triple quadrupole (QqQ) [22].

The conditions of ESI were as follows: 3 kV (capillary voltage), 35 V (cone voltage). Regarding temperature, the ion source temperature was set at 150^0^ C, the pressure of the nitrogen gas (nebulizer) was set at 35 psi, the temperature of drying and sheath gas (N_2_) was 440^◦^C and 350^◦^C, respectively. At 900 L/h and 50 L/h, the drying and sheath gas flows were applied, respectively. The total run time of the analysis was 30 min. The full range acquisition covering 50-1000 m/z was applied to get MS spectra. Regarding automatic MS/MS fragmentation analyses of the parent ions, parent ions masses were selected using the first quadrupole (Q1), parents’ ions were fragmented in the second quadrupole (Q2) through collision-induced dissociation (CID) technique. The energy ramp used was from 30 to 70 eV using collision gas (N_2_). In the negative ion mode, fragmentation of flavonoids and terpene acids was done using collision energy ranging from 20 to 40 eV. Finally, in third quadrupole (Q3), the daughter ions which yielded from the fragmentation were monitored. Daughter ions are related to the molecular structure of the parent ions. The same conditions of chromatography and mass spectrometry described above were applied to MS^n^ experiments. The annotation of metabolites was done by comparing their retention times to that of external standards. Furthermore, our in-house database, data published in literature in addition to phytochemical dictionary of natural products database (CRC), quasi-molecular ions and characteristic MS/MS fragmentation patterns were used for metabolite assignment to get metabolite annotation with a high confidence level.

### Statistical analysis

For semi-quantitative analysis, one-way analysis of the variance (ANOVA) was used with the program SPSS 26.0 (SPSS Inc., Chicago, IL, USA). For metabolomics multivariate statistical data analysis, the SIMCA 14 program (Umetrics, Malmo, Sweden) was used. For MS data analysis, Metaboanalyst 4.0 (http://www.metaboanalyst.ca/), a web-based metabolomics data processing application, was used to create hierarchical cluster analysis heat maps, volcano plots, coefficient plots, and unsupervised self-organizing maps (SOM).

## Results

### Annotation of secondary metabolites in the tested extracts

**Fig. 1:**
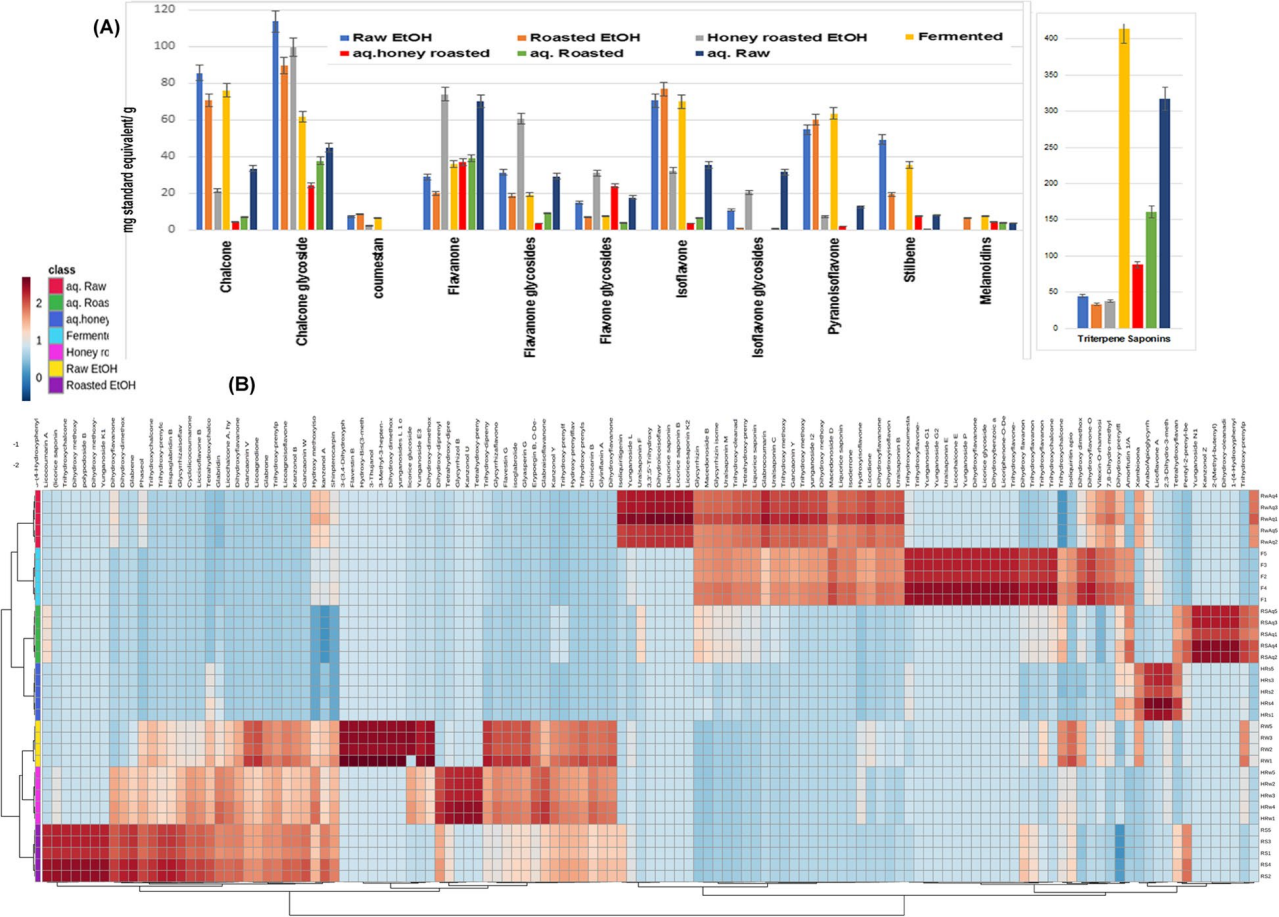
Relative quantitation of the total content of different chemical classes annotated in licorice samples expressed as mg Equivalents (Eq.)/ 100 g dry weight (**A**). Hierarchical analysis heat maps of all annotated constituents in the tested licorice samples. Brick red and blue indicate higher and lower abundances, respectively (**B**)

The UPLC-QqQ-MS/MS analysis (Figure S1) of raw, roasted, honey roasted and fermented licorice roots samples revealed many metabolites belonging to distinct chemical classes. A total of 133 chromatographic peaks were designated in the different samples, with saponins, flavonoids, chalcones, pterocarpans and coumarins being the most abundant groups (Table 1; Fig. 1). A significant amount of structural data was gathered by evaluating the chromatographic behavior of the annotated compounds, as well as related fragmentation pathways already published in the literature. Table 1 displays the full list of annotated compounds and their structural data, including retention time, protonated molecules [M + H]^+^, deprotonated molecules [M–H]^–^, diagnostic MS fragmentation patterns, and molecular formulas. Numbers were allocated to the compounds depending on the order in which they were eluted.

**Table 1:** Metabolites annotated in the different licorice root samples extracts using UPLC-MS/MS in positive and negative ionization modes

ID	Rt	Synonym	Molecular formula	Class	Molecular weight	Ion type	MS^2^ Fragments
1	1.14	Phenol, dimethoxy-4-(2-propenyl)	_14_O_11_H_3_C	Melanoidin	194	[M-H]^−^	175 (M-H-H_2_O), 162 (M-H-OCH_3_), 131 (M-H-2 OCH_3_), 152 (M-H-C_3_H_5_)
2	1.21	Pyran-4-one, dihydro-dihydroxy-methyl	_7_O_6_H_4_C	Melanoidin	143	[M-H]^−^	124 (M-H_2_O), 127 (M-CH_3_), 114 (M-CO)
3	1.36	Furanmethanol	_6_O_5_H_2_C	Melanoidin	98	[M-H]^−^	79 (M-H-H_2_O), 69 (M-H-CO), 65 (M-H-CH_3_OH)
4	1.37	Phenethylamine, methyl-N-vanillyl	_21_O_2_N_17_HC	Melanoidin	271	[M + H]^+^	254 (M + H-H_2_O), 241 (M + H-OCH_3_), 153 (M + H-C_9_H_11_)
5	1.4	Dihydroxyacetophenone	_8_O_8_H_3_C	Melanoidin	152	[M-H]^−^	137 (C_7_H_5_O_3_), 109 (C_6_H_5_O_2_)
6	1.42	Dihydro-methylpyrrolopyrimidinone	C_8_H_10_N_2_O	pyrimidine derivative	150	[M + H]^+^	136 (M + H-CH_3_), 123 (M + H-CO)
7	1.45	Dihydro-3-methylfuran	_8_O_5_HC	Melanoidin	84	[M-H]^−^	68 (M-H-CH_3_), 55 (M-H-CO), 54 (C_4_H_6_)
8	1.47	2-(Methyl-butenyl)-5-(phenylethyl)-benzenediol	_22_O_19_H_2_C	Resorcinol	282	[M-H]^−^	212(M-H- C_5_H_9_), 109 (M-H- C_13_H_16_), 80 (C_6_H_8_), 68 (C_5_H_8_)
9	1.49	Pentyl-2-prenyl-benzenediol-3-Me ether	_26_O _17_H_2_C	Resorcinol	262	[M-H]^−^	192 (M-H-C_5_H_9_), 123 (M-H-C_10_H_20_), 80 (C_6_H_8_), 68 (C_5_H_8_)
10	1.52	Dihydroxybenzoic acid- Xylopyranosyl ester	_14_O_12_H_8_C	Phenolic acid ester	286	[M-H]^−^	267(M-H-H_2_O), 153 (M-H-xylopyranose), 109 (M-H-xylopyranose-CO_2_)
11	1.546	Hydroxy-Bis(3-methyl-2-butenyl)-5-(2-phenylethenyl)-benzenediol	C_24_H_28_O_3_	Stilbene	364	[M + H]^+^	227 (M + H-2 isoprenyl unit), 296 (M + H-isoprenyl unit)
12	1.56	1-(4-Hydroxyphenyl)-3-(7-methoxybenzofuran-6-yl) propanone	_16_O_18_H_4_C	Aliphatic ketone	296	[M-H]^−^	202 (C_12_H_10_O_3_), 174 (C_11_H_10_O_2_), 120 (C_7_H_4_O_2_)
13	1.57	3-Methyl-3-hepten-2-one	_14_O_8_HC	Aliphatic ketone	126	[M + H]^+^	112 (C_7_H_12_O), 84 (C_6_H_12_)
14	1.69	3-(4-Hydroxyphenyl)-phenyl-propenone	C_15_H_12_O_2_	Chalcone	224	[M-H]^−^	146 (X2), 118 (Y2), 104 (Y1), 76 (X1) (Figure S2)
15	2.03	Thujanol	C_10_H_18_O	Monoterpene alcohol	154	[M + H]^+^	137 (M + H-H_2_O), 140 (M + H-CH_3_)
16	2.05	Trihydroxy-4-methoxybibenzyl	C_15_H_16_O_4_	Stilbene	260	[M-H]^−^	241 (M-H-H_2_O), 228 (M-H-OCH_3_)
17	7.74	Dihydroxyflavanone-Di-O-hexoside	C_27_H_32_O_14_	Flavanone glycosides	580	[M + H]^+^	419 (M + H-hexose), 257 (M + H-2 hexose), 239 (M + H-2 hexose -H_2_O), 229 (M + H-2 hexose -CO), 213 (M + H -2 hexose -CO_2_), 137 (^1,3^ A^+^), 163 (M + H -2 hexose-ring B)
18	8.1	Amorfrutin 1/A^a^	C_21_H_24_O_4_	Stilbene	340	[M-H]^−^	225 (C_15_H_13_O_2_), 295 (M-H-CO_2_), 270 (M-H-isoprenyl unit)
19	9.1	Dihydroxyflavanone-O-hydroxypropanoyl hexoside	C_24_H_26_O_11_	Flavanone glycosides	490	[M-H]^−^	257(M-H-Hydroxypropanoyl hexose), 135 (^1,3^ A^−^), 161((M-H-Hydroxypropanoyl hexose-ring B)
20	9.86	3-(3,4-Dihydroxyphenyl)-2-propenoic acid- docosyl ester	_52_O_31_H_4_C	Phenolic acid ester	488	[M + H]^+^	471 (M + H-H_2_O), 180 (M + H-docosanol), 136 (M + H-docosanol-CO_2_), 118 (M + H-docosanol-CO_2_ -H_2_O)
21	9.93	Tetrahydroxyflavan-O-pentoside	C_20_H_22_O_9_	Flavane glycosides	406	[M-H]^−^	273 (M-H-pentose), 295 (M-H-pentose-ring B)
22	10	Dihydroxy dimethoxy-O-hexoside	C_23_H_24_O_11_	Isoflavone glycosides	476	[M + H]^+^	315 (M + H-hexose), 297 (M + H -hexose -H_2_O), 287 (M + H -hexose -CO), 271 (M + H-hexose-CO_2_), 285 (M + H-hexose − 2CH_3_), 167 (^1,3^ A^+^), 149 (^1,3^B^+^)
23	10.27	Trihydroxychalcone-O -Rhamnopyranosyl glucopyranoside]	C_27_H_32_O_13_	Chalcone glycoside	564	[M + H]^+^	419 (M + H-rhamnose), 257 (M + H-rhamnoglucose), 137 (ring A)
24	10.28	Dihydroxyflavanone-O-rutinoside^a^	C_27_H_32_O_13_	Flavanone glycosides	564	[M-H]^−^	257 (M-H- rhamnoglucose) ,135 (^1,3^ A^−^), 161 (M-H-ring B)
25	10.59	Dihydroxyflavone-O-pentosyl hexoside	C_26_H_28_O_13_	Flavone glycosides	548	[M-H]^−^	253 (M-H-pentohexose), 135 (^1,3^ A^−^), 120 (^1,3^B^−^), 235 (M-H- pentohexose -H_2_O), 209 (M-H- pentohexose- CO_2_)
26	10.66	Trihydroxyflavone-O-Rhamnopyranoside (afzelin) ^a^	C_21_H_20_O_10_	Flavone glycosides	432	[M + H]^+^	287 (M + H-rhamnose), 153 (^1,3^ A^+^), 122 (^1,3^B^+^), 269 (M + H-rhamnose- H_2_O), 243(M + H-rhamnose -CO_2_)
27	10.9	Dihydroxyflavanone-O-pentosyl hexoside	C_26_H_30_O_13_	Flavanone glycosides	550	[M-H]^−^	255(M-H- pentohexose), 135 (^1,3^ A^−^), 161(M-H- pentohexose -ring B)
28	11.05	Isoliquiritin apioside^a^	C_26_H_30_O_13_	Chalcone glycoside	550	[M-H]^−^	255 (M-H- apioglucose), 135 (ring A), 119 (ring B)
29	11.15	Dihydroxy methoxy flavanone-O-hexoside	C_22_H_24_O_9_	Flavanone glycosides	432	[M-H]^−^	269 (M-H-hexose), 251 (M-H-hexose H_2_O), 241 (M-H-hexose-CO), 225 (M-H-hexose-CO_2_), 137 (^1,3^ A^−^) ,161 (M-H-hexose-ring B)
30	11.21	Vitexin-O-rhamnoside^a^	C_21_H_20_O_10_	Flavone-C-glycosides	578	[M-H]^−^	431 (M-H- rhamnose), 341 (Cross link cleavage in glucose unit), 311 (Cross link cleavage in glucose unit)
31	11.46	Dihydroxy flavanone-O-hexoside	C_21_H_22_O_9_	Flavanone glycosides	418	[M + H]^+^	257 (M + H-hexose), 239 (M + H-hexose-H_2_O), 229 (M + H-hexose-CO), 213 (M + H-hexose- CO_2_),137 (^1,3^ A^+^), 163 (M + H-hexose -ring B)
32	11.93	Yunganoside G1	C_48_H_74_O_21_	Triterpene Saponin (oleanane type)	987	[M-H]^−^	840 (M-H-rhamnose), 489 (M-H-di-glucouronic residue- rhamnose),470 ((M-H-di-glucouronic residue- rhamnose-H_2_O), 497 (rhamnose di-glucouronic residue -H), 458 (M-H- di-glucouronic residue- rhamnose-CH_2_O), 351 (di-glucouronic residue -H)
33	12.12	Trihydroxyflavanone-O-pentosyl hexoside	C_26_H_30_O_14_	Flavanone glycosides	566	[M-H]^−^	271 (M-H-pentohexose),155 (^1,3^ A^−^), 177 (M-H-pentohexose-ring B)
34	12.43	Trihydroxyflavanone-O-hexoside	C_21_H_22_O_10_	Flavanone glycosides	434	[M + H]^+^	273 (M + H-hexose), 169 (^1,3^ A^+^), 195 (M + H-hexose-ring B)
35	12.58	Trihydroxychalcone-O-Apiofuranosyl glucopyranoside	C_26_H_30_O_13_	Chalcone glycoside	550	[M + H]^+^	419 (M + H-apiose), 257 (M + H- apioglucose), 137 (ring A)
36	12.6	Uralsaponin E^a^	C_42_H_60_O_17_	Triterpene Saponin (oleanane type)	836	[M-H]^−^	659 (M-H-glucouronic residue), 483 (M-H– di-glucouronic residue), 465 (M-H-di-glucouronic residue- H_2_O), 437 (M-H-di-glucouronic residue- H_2_O- CH_2_O), 447 (M-H-di-glucouronic residue − 2H_2_O), 176 (glucouronic residue)
37	12.74	Trihydroxyflavone-glucoside^a^	C_21_H_18_O_11_	Flavone glycosides	446	[M-H]^−^	269 (M-H-glucose), 105 (^1,3^B^−^), 167 (^1,3^ A^−^), 265 (M-H-glucose-H_2_O), 239 (M-H-glucose-CO_2_)
38	12.83	Dihydroxyisoflavone-dimethoxy-O-hydroxyphenyl)propanoyl-glucoside	C_32_H_32_O_13_	Isoflavone glycosides	624	[M-H]^−^	309 (M-H-hydroxy phenyl propanoyl glucose), 291 (M-H- hydroxy phenyl propanoyl glucose -H_2_O), 281 (M-H- hydroxy phenyl propanoyl glucose -CO), 265 (M-H- hydroxy phenyl propanoyl glucose -CO_2_), 165 (^1,3^ A^−^), 147 (^1,3^B^−^)
39	13.01	licorice glucoside D1^a^	C_35_H_36_O_15_	Flavanone glycosides	696	[M-H]^−^	255 (M-H-coumaroyl apioglucose),135 (^1,3^ A^−^), 119 (ring B)
40	13.05	Trihydroxychalcone-O-Hydroxycinnamoyl-apiofuranosyl-glucopyranoside	C_35_H_36_O_15_	Chalcone glycoside	696	[M-H]^−^	401 (M-H- apioglucose), 255 (M-H-hydroxycinnamoyl apioglucose), 135 (ring A)
41	13.12	Hydroxy methoxy isoflavone-O-hexoside	C_22_H_22_O_9_	Isoflavone glycosides	430	[M-H]^−^	269 (M-H-hexose), 251(M-H-hexose-H_2_O), 241 (M-H-hexose -CO), 225 (M-H-hexose -CO_2_), 137 (^1,3^ A^−^), 133 (^1,3^B^−^)
42	13.2	Licorice glycoside C1 4’,7-Dihydroxyflavanone; (S)-form, 4’-O-[4-Hydroxy-3-methoxycinnamoyl-(→5)-β-D-apiofuranosyl-(1→2)-β-D-glucopyranoside]	C_36_H_38_O_16_	Flavanone glycosides	726	[M-H]^−^	710 (M-H-CH_3_), 255 (M-H- Hydroxy methoxycinnamoyl apioglucose), 135 (^1,3^ A^−^), 119 (^1,3^B^−^)
43	13.34	Yunganosides L 1 or J1	C_48_H_72_O_20_	Triterpene Saponin (oleanane type)	969	[M-H]^−^	950 (M-H- H_2_O), 833 (M-H- C_4_H_6_O_5_), 645 (M-H- C_4_H_6_O_5_-C_8_H_12_O_5_), 497 (rhamnose di-glucouronic residue–H), 453(M-H -di-glucouronic rhamnose -H_2_O), 176 (glucouronic residue), 162 (glucose residue)
44	13.42	Liquorice saponin F3	C_48_H_72_O_19_	Triterpene Saponin (oleanane type)	953	[M-H]^−^	497 (rhamnose di-glucouronic residue -H), 436 (M-H-di-glucouronic-rhamnose-H_2_O), 418(M-H-di-glucouronic-rhamnose − 2 H_2_O), 454 (M-H-di-glucouronic-rhamnose), 176 (glucouronic residue)
45	13.44	Uralsaponin F^a^	C_44_H_64_O_19_	Triterpene Saponin (oleanane type)	896	[M-H]^−^	719 (M-H-glucouronic residue), 543 (M-H-di-glucouronic residue), 525 (M-H-di-glucouronic residue-H_2_O), 507 (M-H-di-glucouronic residue- 2 H_2_O), 497 (rhamnose di-glucouronic residue -H), 495(M-H-di-glucouronic residue-H_2_O-CH_2_O) 465 (M-H- di-glucouronic residue- H_2_O-acetyl residue), 447(M-H- di-glucouronic residue − 2H_2_O-acetyl residue)
46	13.52	Liquorice saponin J2^a^	C_42_H_64_O_16_	Triterpene Saponin (oleanane type)	824	[M-H]^−^	453 (M-H-di-glucouronic residue -H_2_O), 471(M-H- di-glucouronic residue), 647(M-H-glucouronic residue)
47	13.83	Tetrahydroxychalcone-Me ether	C_16_H_14_O_5_	Chalcone	286	[M-H]^−^	270 (M-H-CH_3_), 151 (ring A), 133 (ring B)
48	13.94	Trihydroxychalcone-O-Hydroxy-methoxycinnamoyl-apiofuranosyl glucopyranoside	C_36_H_38_O_16_	Chalcone glycoside	726	[M + H]^+^	433 (M + H- apioglucose), 257 (M + H-hydroxy methoxycinnamoyl apioglucose), 137 (ring A)
49	14.03	Yunganoside K1	C_48_H_72_O_21_	Triterpene Saponin (oleanane type)	985	[M-H]^−^	497 (rhamnose di-glucouronic residue–H), 486 (M-H- di-glucouronic-rhamnose), 176 (glucouronic residue)
50	14.3	Polypodoside B	C_39_H_62_O_13_	Triterpene Saponin (cholestane type)	738	[M + H]^+^	593 (M + H-rhamnose), 577(M + H-glucose), 431 (M + H-glucose-rhamnose)
51	14.55	Yunganoside G2^a^	C_42_H_64_O_17_	Triterpene Saponin (oleanane type)	840	[M-H]^−^	777 (M-H-H_2_O-CO_2_), 487 (M-H-di-glucouronic), 469 (M-H-di-glucouronic-H_2_O), 351(di-glucouronic residue-H),
52	14.68	Dihydroxy-dimethoxyflavone	C_17_H_14_O_6_	Flavone	314	[M + H]^+^	297 (M + H-H_2_O), 271 (M + H-CO_2_), 153 (^1,3^ A^+^), 166 (^1,3^B^+^)
53	15.03	Uralsaponin M	C_44_H_64_O_18_	Triterpene Saponin (oleanane type)	880	[M-H]^−^	703 (M-H-glucouronic residue), 527(M-H-di-glucouronic residue)
54	15.22	(Licorice saponin G2) 24-Hydroxyglycyrrhizin^a^	C_42_H_62_O_17_	Triterpene Saponin (oleanane type)	838	[M + H]^+^	821(M + H-H_2_O), 777 (M + H-H_2_O-CO_2_), 663 (M + H- glucouronic residue), 351(di-glucouronic residue -H)
55	15.24	Yunganoside P^a^	C_42_H_60_O_17_	Triterpene Saponin (oleanane type)	836	[M-H]^−^	483 (M-H- di-glucouronic residue), 465 (M-H- di-glucouronic residue -H_2_O), 434 (M-H- di-glucouronic residue -H_2_O-OCH_3_), 176 (glucouronic residue)
56	15.43	Dihydroxyflavanone-O-Indolylcarbonyl-pentosyl hexoside	C_35_H_35_NO_14_	Flavanone glycoside	693	[M-H]^−^	400 (M-H-pentohexose), 241(M-H- indolylcarbonyl pentohexose), 135 (^1,3^ A^−^), 161 (M-H- indolylcarbonyl pentohexose -ring B)
57	15.54	Yunganoside N1	C_42_H_64_O_14_	Triterpene Saponin (oleanane type)	792	[M + H]^+^	617(M + H-glucouronic residue), 441((M + H-di-glucouronic residue), 353 (di-glucouronic residue + H),
58	15.8	Licorice saponin B2 or Isomer of licorice saponin B2	C_42_H_64_O_15_	Triterpene Saponin (oleanane type)	808	[M-H]^−^	789(M-H-H_2_O), 613(M-H-glucouronyl residue-H_2_O), 351(di-glucouronic residue -H)
59	16.45	Dihydroxy methoxy isoflavane	C_16_H_16_O_4_	Isoflavane	272	[M-H]^−^	258 (M-H-CH_3_), 123 (ring A), 149 (M-H-ring B)
60	16.68	Dihydroxy methoxy-prenylisoflavone	C_21_H_20_O_5_	Isoflavone	352	[M-H]^−^	333 (M-H-H_2_O), 323 (M-H-CO), 307 (M-H-CO_2_), 336 (M-H-CH_3_), 281(M-H- isoprenyl unit), 117 (^1,3^B^−^), 218 (^1,3^ A^−^)
61	17.35	Licoricesaponin K2	C_42_H_62_O_16_	Triterpene Saponin (oleanane type)	822	[M + H]^+^	647(M + H- glucouronic residue), 471 (M + H- di-glucouronic residue), 453(M + H- di-glucouronic residue - H_2_O)
62	17.65	Macedonoside D	C_42_H_60_O_16_	Triterpene Saponin (oleanane type)	820	[M-H]^−^	467(M-H-di-glucouronic residue), 449(M-H- di-glucouronic residue -H_2_O)
63	18.14	Flavestin B^a^	_20_O_19_H_2_C	Resorcinol	280	[M + H]^+^	212 (M + H-isoprenyl unit), 111 (M + H-isoprenyl unit- C_8_H_5_), 82 (C_6_H_10_), 70 (C_5_H_10_), 54 (C_4_H_6_)
64	18.2	Macedonoside B	C_42_H_62_O_17_	Triterpene Saponin (oleanane type)	838	[M-H]^−^	819 (M-H-H_2_O), 485 (M-H- diglucouronic residue), 351(diglucouronic residue -H)
65	18.35	Uralsaponin B	C_42_H_62_O_16_	Triterpene Saponin (oleanane type)	822	[M + H]^+^	647 (M + H − glucouronic residue), 471(M-H- di-glucouronic residue), 351(di-glucouronic residue-H)
66	18.87	Isoliquiritigenin^a^	C_15_H_12_O_4_	Chalcone	256	[M-H]^−^	135 (ring A), 119 (ring B), 93 (ring B fragment)
67	19.1	Glycyrrhizin^a^	C_42_H_62_O_16_	Triterpene Saponin (oleanane type)	822	[M-H]^−^	803 (M -H-H_2_O), 759 (M-H-H_2_O-CO_2_), 645(M -H- glucouronic residue), 351 (di-glucuronic acid residue -H)
68	19.5	Trihydroxycoumestan-Me ether	C_16_H_10_O_6_	Coumestan	298	[M + H]^+^	268 (M + H- OCH_3_), 281(M + H-H_2_O)
69	19.59	Dihydroxy dimethoxyisoflavone	C_17_H_14_O_6_	Isoflavone	314	[M-H]^−^	297 (M-H-H_2_O), 287 (M-H-CO), 271 (M-H-CO_2_), 285 (M-H-2 CH_3_), 167 ((^1,3^ A^−^), 149 (^1,3^B^−^)
70	19.62	Hydroxy methoxyisoflavone	C_16_H_12_O_4_	Isoflavone	268	[M-H]^−^	251 (M-H-H_2_O), 241 (M-H-CO), 225 (M-H-CO_2_), 137 (^1,3^ A^−^), 133 ((^1,3^B^−^)
71	19.66	Licodione^a^	C_15_H_12_O_5_	Chalcone	272	[M-H]^−^	162 (X2), 136 (Y1), 134 (Y2),108 (X1) (Figure S2)
72	19.81	Licoriphenone-O-De-Me	_22_O_20_H_6_C	Aliphatic ketone	358	[M + H]^+^	290 (M + H-isoprenyl unit), 250 (C_14_H_18_O_4_), 222 (C_13_H_18_O_3_), 138 (C_7_H_6_O_3_)
73	19.9	Dihydroxy-oleanadienoic acid-diglycoside	C_42_H_62_O_14_	Triterpene Saponin (oleanane type)	790	[M-H]^−^	447 (M-H-2 hexose − 2 H_2_O), 465 (M-H-2 hexose)
74	20.26	Xambioona	C_25_H_24_O_4_	Flavanone	388	[M-H]^−^	369 (M-H-H_2_O), 359 ((M-H-CO), 343 (M-H-CO_2_), 201 (^1,3^ A^−^), 227 (M-H-ring B)
75	20.44	Trihydroxy-oleanadienoic acid-diglycoside	C_42_H_64_O_15_	Triterpene Saponin (oleanane type)	808	[M-H]^−^	483 (M-H-2 hexose), 465 (M-H-2 hexose -H_2_O), 447 (M-H-2 hexose − 2 H_2_O)
76	20.76	Glycyrrhizin isomer	C_42_H_62_O_16_	Triterpene Saponin (oleanane type)	822	[M-H]^−^	803(M-H-H_2_O), 759 (M-H-H_2_O-CO_2_), 645 (M- H-glucouronic residue), 351(di-glucouronic residue - H)
77	20.94	Tetrahydroxy-prenylchalcone	C_20_H_20_O_5_	Chalcone	340	[M-H]^−^	270 (M-H-isoprenyl unit), 203 (ring B), 135 (ring A)
78	21.07	Trihydroxy methoxy-prenylisoflavone	C_21_H_20_O_6_	prenyl Isoflavone	368	[M-H]^−^	351 (M-H-H_2_O), 341 (M-H-CO), 325 (M-H-CO_2_), 354 (M-H-CH_3_), 298 (M-H-isoprenyl unit), 149 (^1,3^B^−^), 222 (M-H- ring A)
79	21.1	Trihydroxy-prenylflavanone	C_20_H_20_O_5_	Flavanone	340	[M + H]^+^	323 (M + H-H_2_O), 313 (M + H-CO), 297 (M + H-CO_2_), 272 (M + H-isoprenyl unit), 221 (^1,3^ A^−^), 247 (M + H-ring B)
80	21.2	Gancaonin Y^a^	C_21_H_22_O_4_	Isoflavane	338	[M + H]^+^	324(M + H-CH_3_), 123 (^1,3^ A^+^) ,149 (M + H-ring B)
81	21.3	Glabrocoumarin^a^	C_20_H_16_O_5_	Coumarin	336	[M + H]^+^	163 (C_9_H_7_O_3_), 119 (C_8_H_7_O)
82	21.6	Gancaonin V	C_19_H_20_O_4_	Prenylated dihydrophenanthrene	312	[M + H]^+^	244 (M + H-isoprenyl unit), 285 (M + H-CO)
83	21.74	Arabo/Apioglycyrrhizin	C_41_H_62_O_14_	Triterpene Saponin (oleanane type)	778	[M-H]^−^	715(M-H H_2_O-CO_2_), 645(M-H-arabinose)
84	21.91	Uralsaponin C^a^	C_42_H_64_O_16_	Triterpene Saponin (oleanane type)	824	[M-H]^−^	647(M-H- glucouronic residue), 471[M-H-di- glucouronic residue], 453[M-H–di- glucouronic residue -H_2_O], 435(M-H–di-glucouronic residue − 2H_2_O), 417[M-H– di-glucouronic residue − 3H_2_O)
85	21.96	Tetrahydroxy-prenylflavanone	C_20_H_18_O_6_	Flavanone	354	[M + H]^+^	337 (M + H-H_2_O), 327 (M + H-CO), 311 (M + H-CO_2_), 286 (M + H-isoprenyl unit), 221 (^1,3^ A^+^), 245(M + H-ring B)
86	22.01	Chiricanin B	C_19_H_20_O_3_	Stilbene	296	[M + H]^+^	279 (M + H- H_2_O), 205 (C_12_H_13_O_3_), 89 (C_7_H_5_)
87	22.54	Liquorice saponin C2	C_42_H_62_O_15_	Triterpene Saponin (oleanane type)	806	[M-H]^−^	787(M-H-H_2_O), 629 (M- H- glucouronic residue), 351(di-glucouronic residue -H)
88	22.74	Isoderrone	C_20_H_16_O_5_	Pyranoisoflavone	336	[M + H]^+^	319 (M + H-H_2_O), 309 (M + H-CO), 293 (M + H-CO_2_), 322 (M + H-CH_3_), 153 (^1,3^ A^+^), 201 (^1,3^B^+^)
89	22.8	Licochalcone E^a^	C_21_H_22_O_4_	Chalcone	338	[M + H]^+^	324 (M + H-CH_3_), 270 (M + H-isoprenyl unit), 231 (X2-CH_3_), 203 (Y2-CH_3_), 122 (Y1), 94 (C_6_H_6_O X1) (Figure S2)
90	22.9	Tetrahydroxy-diprenylflavanone	C_25_H_28_O_6_	Flavanone	424	[M-H]^−^	405 (M-H-H_2_O), 395 (M-H-CO), 379 (M-H-CO_2_), 285 (M-H-2 isoprenyl unit), 151 (^1,3^ A^−^), 181(M-H-ring B)
91	23.03	Trihydroxy methoxy isoflavone	C_16_H_12_O_6_	isoflavone	300	[M + H]^+^	283 (M + H-H_2_O), 273 (M + H-CO), 257 (M + H-CO_2_), 286 (M + H-CH_3_), 153 (^1,3^ A^+^), 149 (^1,3^B^+^)
92	23.1	Yunganoside I2	C_42_H_64_O_15_	Triterpene Saponin (oleanane type)	808	[M-H]^−^	745 (M- H_2_O-CO_2_-H), 631(M-H- glucouronic residue), 613(M-H-glucouronic residue -H_2_O), 351(di-glucouronic residue -H)
93	23.26	Trihydroxy-prenylchalcone-Me ether	C_21_H_22_O_4_	Chalcone	338	[M-H]^−^	268 (M-H-isoprenyl unit), 229 (X2-CH_3_), 201 (Y-CH_3_), 120 (Y1), 92 (X1) (Figure S2)
94	23.5	Dihydroxy-prenylflavanone	C_20_H_20_O_4_	flavanone	324	[M + H]^+^	307 (M + H-H_2_O), 297 (M + H-CO), 281 (M + H-CO_2_), 256 (M + H-isoprenyl unit), 220 (^1,3^ A^+^), 247 (M + H-ring B)
95	24.13	Phaseol^a^	C_20_H_16_O_5_	coumestan	336	[M-H]^−^	250 (M-H- C_4_H_8_-CHO), 251(M-H- C_4_H_8_-CO), 279 (M- H-C_4_H_8_), 280(M-H-C_4_H_7_)
96	24.51	kanzonol A^a^	C_20_H_20_O_5_	Chalcone	340	[M-H]^−^	270 (M-H-isoprenyl unit), 108 (X1), 230 (X2), 136 (Y1), 202 (Y2) (Figure S2)
97	24.624	Glycyrrhizol B^a^	C_21_H_18_O_5_	Pterocarpan	350	[M-H]^−^	212 (C_14_H_12_O_2_), 188 (C_12_H_12_O_2_), 174 (C_10_H_6_O_3_)
98	24.7	Yunganoside L	C_36_H_54_O_10_	Triterpene Saponin (oleanane type)	646	[M + H]^+^	471 (M + H-glucouronic residue), 176 (glucouronic residue)
99	24.86	Gancaonin W^a^	C_21_H_20_O_6_	isoflavone	368	[M-H]^−^	298 (M-H-isoprenyl unit), 339(M-H-CO), 323(M-H-CO_2_)
100	25.19	Trihydroxy-prenylstilbene	C_19_H_20_O_3_	Stilbene	296	[M-H]^−^	189 (C_12_H_13_O_2_), 226 (M-H- isoprenyl unit)
101	25.3	Licoisoflavone B^a^	C_20_H_16_O_6_	Pyranoisoflavone	352	[M-H]^−^	333 (M-H-H_2_O), 323 (M-H-CO), 307 (M-H-CO_2_), 336 (M-H-CH_3_), 151 (^1,3^ A^−^), 215 (^1,3^B^−^)
102	25.44	Kanzonol U	C_19_H_16_O_4_	2-arylbenzofuran flavonoids	308	[M-H]^−^	289 (M-H-H_2_O), 263 (M-H-CO_2_)
103	25.65	Flavestin G	C_19_H_20_O_2_	Stilbene	280	[M-H]^−^	189 (C_12_H_13_O_2_), 210 (M-H-isoprenyl unit)
104	25.86	Licoagrodione^a^	C_20_H_20_O_6_	Stilbene	356	[M-H]^−^	150 (C_8_H_6_O_3_), 204 (C_12_H_12_O_3_)
105	26.14	Licoagroisoflavone^a^	C_20_H_16_O_5_	isoflavone	336	[M-H]^−^	317 (M-H-H_2_O), 307 (M-H-CO), 291 (M-H-CO_2_), 117 (^1,3^B^−^), 201(^1,3^ A^−^)
106	26.23	Trihydroxy-prenylpterocarpan-Me ether	C_21_H_22_O_5_	Pterocarpan	354	[M-H]^−^	284 (M-H-isoprenyl unit), 244 (C_15_H_16_O_3_), 146 (C_9_H_6_O_2_), 122 (C_7_H_6_O_2_)
107	26.43	Trihydroxy-prenylpterocarpan-Didehydro-Me ether	C_21_H_20_O_5_	Pterocarpan	352	[M-H]^−^	282 (M-H-isoprenyl unit), 242 (C_15_H_14_O_3_), 122 (C_7_H_6_O_2_)
108	26.59	Glycyrrhizaisoflavone C	C_21_H_20_O_6_	isoflavone	368	[M-H]^−^	349 (M-H-H_2_O), 339 (M-H-CO), 323 (M-H-CO_2_),135 (^1,3^ A^−^), 245 (^1,3^B^−^)
109	26.88	Glycyrrhizaflavonol A	C_20_H_18_O_7_	Pyranoflavonol	370	[M-H]^−^	341(M-H-CO), 354 (M-H-CH_3_), 325 (M-H-CO_2_), 351 (M-H-H_2_O), 151 (ring A)
110	26.97	Glabridin^a^	C_20_H_20_O_4_	Pyranoisoflavan	324	[M-H]^−^	295 (M-H-CO), 279 (M-H-CO_2_), 187 (^1,3^ A^−^), 135 (^1,3^B^−^)
111	27.26	Glabrone^a^	C_20_H_16_O_5_	Pyranoisoflavone	335	[M-H]^−^	319 (M-H-CH_3_), 290 (M-H-CO_2_), 136 (^1,3^ A^−^), 199 (^1,3^B^−^), 213 (^1,4^B^−^-H_2_O)
112	27.32	Hydroxy-prenylflavanone	C_20_H_20_O_3_	flavanone	308	[M + H]^+^	291(M + H-H_2_O), 281(M + H-CO), 265 (M + H-CO_2_), 240 (M + H-isoprenyl unit), 204 (^1,3^ A^+^), 231(M + H-ring B)
113	27.82	Dihydroxy-diprenylflavanone	C_25_H_28_O_4_	flavanone	392	[M + H]^+^	375 (M + H-H_2_O), 365 (M + H-CO), 349 (M + H-CO_2_), 255 (M + H-2 isoprenyl unit), 204 (^1,3^ A^+^), 231(M + H-ring B)
114	28.03	Glyasperin G	C_21_H_24_O_5_	coumestan	356	[M + H]^+^	301 (M + H-C_4_H_8_), 283 (M + H-C_4_H_8_-H_2_O), 191 (C_11_H_11_O_3_)
115	28.13	Cyclolicocoumarone	_20_O_20_H _5_ C	Resorcinol	340	[M-H]^−^	109 (M-H-C_14_H_16_O_3_), 80 (C_6_H_8_), 68 (C_5_H_8_), 52 (C_4_H_4_)
116	28.22	Licoflavone A	C_20_H_18_O_4_	Flavone	322	[M-H]^−^	252 (M-H-isoprenyl unit), 204 (^1,3^ A^−^), 120 (^1,3^B^−^)
117	28.38	Glabraisoflavanone A	C_25_H_28_O_4_	isoflavanone	392	[M-H]^−^	373 (M-H-H_2_O), 348 (M-H-CO_2_), 363 (M-H-CO), 322 (M-H-isoprenyl unit), 204 (^1,3^ A^−^)
118	28.5	Isoglabrolide	C_30_H_44_O_4_	Triterpene Saponin (oleanane type)	468	[M + H]^+^	451(M + H-H_2_O), 425 (M + H-CO_2_), 413 (M + H- C_4_H_8_), 407 (M + H-CO_2_-H_2_O), 395 (M + H-C_4_H_8_-H_2_O)
119	28.7	Licocoumarin A^a^	C_25_H_26_O_5_	Coumarin	406	[M-H]^−^	336 (M-H- isoprenyl unit), 267 (M-H-2 isoprenyl unit), 229 (C_14_H_13_O_3_), 161 (C_9_H_5_O_3_), 117 (C_8_H_5_O)
120	29.17	Kanzonol Z^a^	_26_O_25_H _5_ C	Flavanone	406	[M-H]^−^	387 (M-H-H2O), 377 (M-H-CO), 361 (M-H-CO2), 336 (M-H-isoprenyl unit), 203 (^1,3^ A^−^), 227 (M-H-ring B)
121	29.44	Kanzonol Y	C_25_H_30_O_5_	Chalcone	410	[M-H]^−^	340 (M-H- isoprenyl unit), 271 (M-H- 2 isoprenyl unit), 232 (X2), 204 (Y1), 176 (X1) (Figure S2)
122	29.45	Licochalcone A, 2’-hydroxy	C_21_H_22_O_5_	Chalcone	354	[M-H]^−^	338 (M-H-CH_3_), 284 (M-H- isoprenyl unit), 217 (ring B), 135 (ring A)
123	30.24	Dihydrolicoisoflavone A	C_20_H_20_O_6_	isoflavanone	356	[M-H]^−^	337 (M-H-H_2_O), 327 (M-H-CO), 311 (M-H-CO_2_), 286 (M-H-isoprenyl unit), 151 (^1,3^ A^−^), 202 (^1,3^B^−^)
124	30.95	Kanzonol B	_18_O_20_H_4_C	Chalcone	322	[M + H]^+^	214 (X2), 187 (Y2), 138 (Y1), 110 (X1) (Figure S2)
125	31.24	Glabrene^a^	C_20_H_18_O_4_	Pyranoisoflavene	322	[M-H]^−^	121 (^1,3^ A^−^), 199 (^1,3^B^−^), 306 (M-H-CH_3_), 293 (M-H-CO), 277 (M-H-CO_2_)
126	32.09	Yunganoside E3	C_36_H_52_O_10_	Triterpene Saponin (oleanane type)	644	[M-H]^−^	467 (M-H-glucouronic residue), 176 (glucouronic residue)
127	32.21	Shinpterocarpin^a^	C_20_H_18_O_4_	Pterocarpan	322	[M-H]^−^	212 (C_14_H_12_O_2_), 146 (C_9_H_6_O_2_), 122 (C_7_H_6_O_2_)
128	32.2	Glycyrrhetinic acid	_30_O_46_H_4_C	Triterpene Saponin	469	[M-H]^−^	450 (M-H-H_2_O), 423 (M-H-COOH), 406 (M-H-CO_2_-H_2_O)
129	32.8	Hispaglabridin B	C_25_H_26_O_4_	Pyranoisoflavan	390	[M + H]^+^	347 (M + H-CO_2_), 363 (M + H-CO), 189 (^2,3^B^+^)
130	33.57	Trihydroxy-diprenylisoflavan	C_25_H_30_O	Isoflavan	394	[M + H]^+^	367 (M + H-CO), 351 (M + H-CO_2_), 257 (M + H-2 isoprenyl unit), 191 (^1,3^ A^+^), 215 (M + H- ring B)
131	34.08	Glyinflanin A	C_25_H_28_O_5_	Chalcone	408	[M-H]^−^	338 (M-H- isoprenyl unit), 269 (M-H-2 isoprenyl unit), 230 (X2), 204 (Y1), 202 (Y2), 176 (X1) [Figure S2]
132	34.1	Erypoegin B, O-De-Me	C_20_H_18_O_4_	Pyranoisoflavene	322	[M-H]^−^	137 (^1,3^ A^−^), 183((^1,3^B^−^), 306 (M-H-CH_3_), 293 (M-H-CO), 277 (M-H-CO_2_)
133	34.2	Dihydroxy-dimethoxy prenylisoflavan	C_22_H_26_O_5_	Isoflavan	370	[M + H]^+^	341 (M + H-2 OCH_3_), 302 (M + H-isoprenyl unit), 153 (^1,3^ A^+^), 179 (M + H-ring B)

^a^Compounds identified by comparison to reference standards

### Tracking the effect of roasting and fermentation changes on the chemical profile of licorice roots via UPLC-QqQ-MS/MS analysis in combination with multivariate statistical analysis

Semi quantitation of the annotated compounds was carried out using representative standards of the identified chemical classes; quercetin, glycrrhizic acid, esculetin, licochalcone A, ellagic acid, trans-stilbene, and 7, 12-dimethoxy coumestan. Standard calibration curves were established by plotting peak areas of the standards as the analytical responses against their known concentration. Validation parameters like linearity, limit of detection (LOD) and limit of quantification (LOQ) were assessed based on FDA guidelines on bioanalytical method validation [23] (Table S2). Standard compounds were effectively used to compute the relative quantities of the detected metabolites. Each studied extract’s measured components were reported as mg standard Equivalents/g dry extract Table S3.

As depicted in Fig. 1. Triterpene saponins overwhelmingly dominated the secondary metabolites in the aqueous extracts of fermented, roasted, honey roasted and raw licorice roots samples, with fermented samples showing the highest relative amounts of saponins. Meanwhile, the ethanol extracts of the tested samples showed significant amounts of chalcones and chalcone glycosides followed by isoflavones. Flavanones and flavanone glycosides showed significant accumulation in the ethanol extracts of honey roasted samples while melanoidins were only detected in the ethanol and aqueous extracts of roasted and honey roasted samples.

Semi-quantitative data of annotated compounds was used to create an unsupervised hierarchical heat map for the investigated samples (Fig. 1B). Licoumarin A, trihydroxy chalcone and polypodoside B were only detected in the ethanol extracts of roasted roots while glycyrrhizol B and kanzonol U were detected only in the ethanol extracts of honey roasted roots. Meanwhile the main licorice saponin glycyrrhizin was mainly detected in the aqueous extracts of raw as well as fermented roots and in lesser amounts in the aqueous extracts of roasted and honey roasted samples. Licorice saponins A and B as well as uralsaponin F were only detected in the aqueous extracts of raw root samples while yuanganosides G1 and G2, uralsaponin E, trihydroxy coumestan glycoside and dihydroxy benzoic acid were detected in the fermented root samples only. The resorcinol 2-(Methyl-butenyl)-5-(phenylethyl)-benzenediol as well as the melanoidins phenol, dimethoxy-4-(2-propenyl) and Pyran-4-one, dihydro-dihydroxy-methyl were only detected in the aqueous extracts of honey roasted samples.

**Fig. 2:**
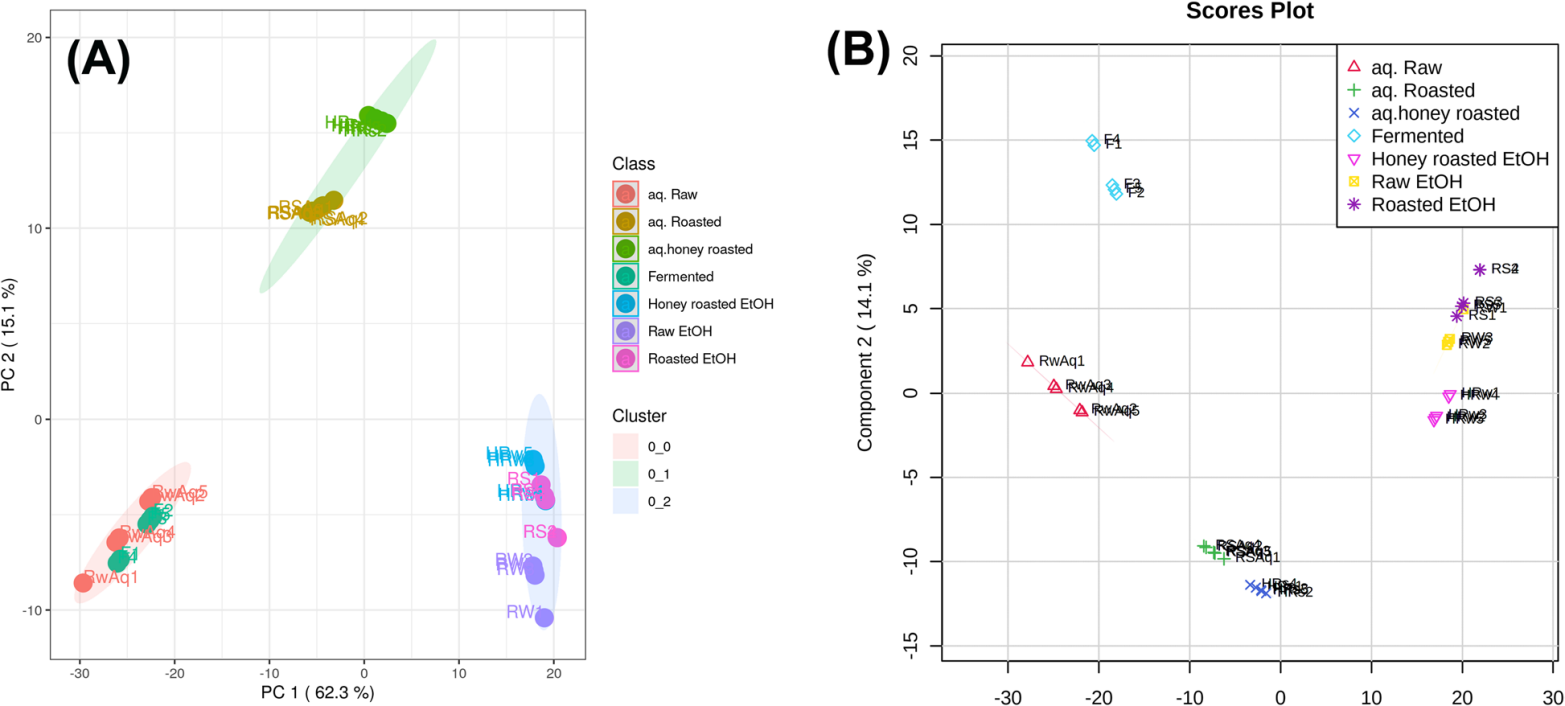
Unsupervised self-organizing map (SOM) of the tested licorice samples (**A**). Orthogonal Projections to Latent Structures Discriminant Analysis (OPLS-DA) score scatter plot (**B**)

MetaboAnalyst 5.0 was used to process the data from the various root samples which were subjected to unsupervised self-organizing map (SOM) analysis, a neural network-based dimensionality reduction approach. Within the samples, PC1 and PC2 explained 62.3% and 15.1% of variation, respectively. As shown in Fig. 2A, samples were divided into three primary clusters, one comprising the aqueous extracts of raw and fermented root samples, the other comprising the aqueous extract of roasted and honey roasted roots and finally a cluster containing the ethanol extracts of raw, roasted and honey roasted samples.

**Fig. 3:**
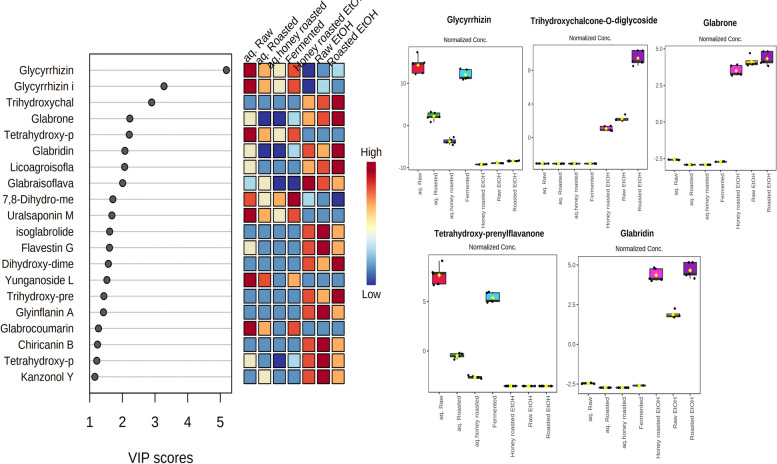
Variables of importance (VIP) plot of the annotated secondary metabolites in the tested licorice samples

The in-between and within-class discrimination of samples using an OPLS-DA (Orthogonal projection to latent structure-discriminant analysis) model based on their phytochemical profiles, as well as markers that chemically distinguish each class was attempted (Fig. 2B). The first component was responsible for 61.3% of sample variation, while the second component was responsible for 14.1%. The correlation coefficient (R_2_ = 0.997) and the redundancy value of cross validation (Q2 = 0.981) values were used to determine the predictability and reliability of the created OPLS-DA model, which demonstrated the model’s predictability and reliability, respectively. The ethanol extracts of the samples showed in-between class discrimination from aqueous extracts while within-class discrimination was observed between the aqueous extracts of fermented and raw and the roasted and honey roasted ones. Variable of importance (VIP) plot (Fig. 3) showed the main chemical features responsible for the discrimination of samples.

### Determination of discriminatory metabolites between roasted, honey roasted and raw licorice roots samples

**Fig. 4:**
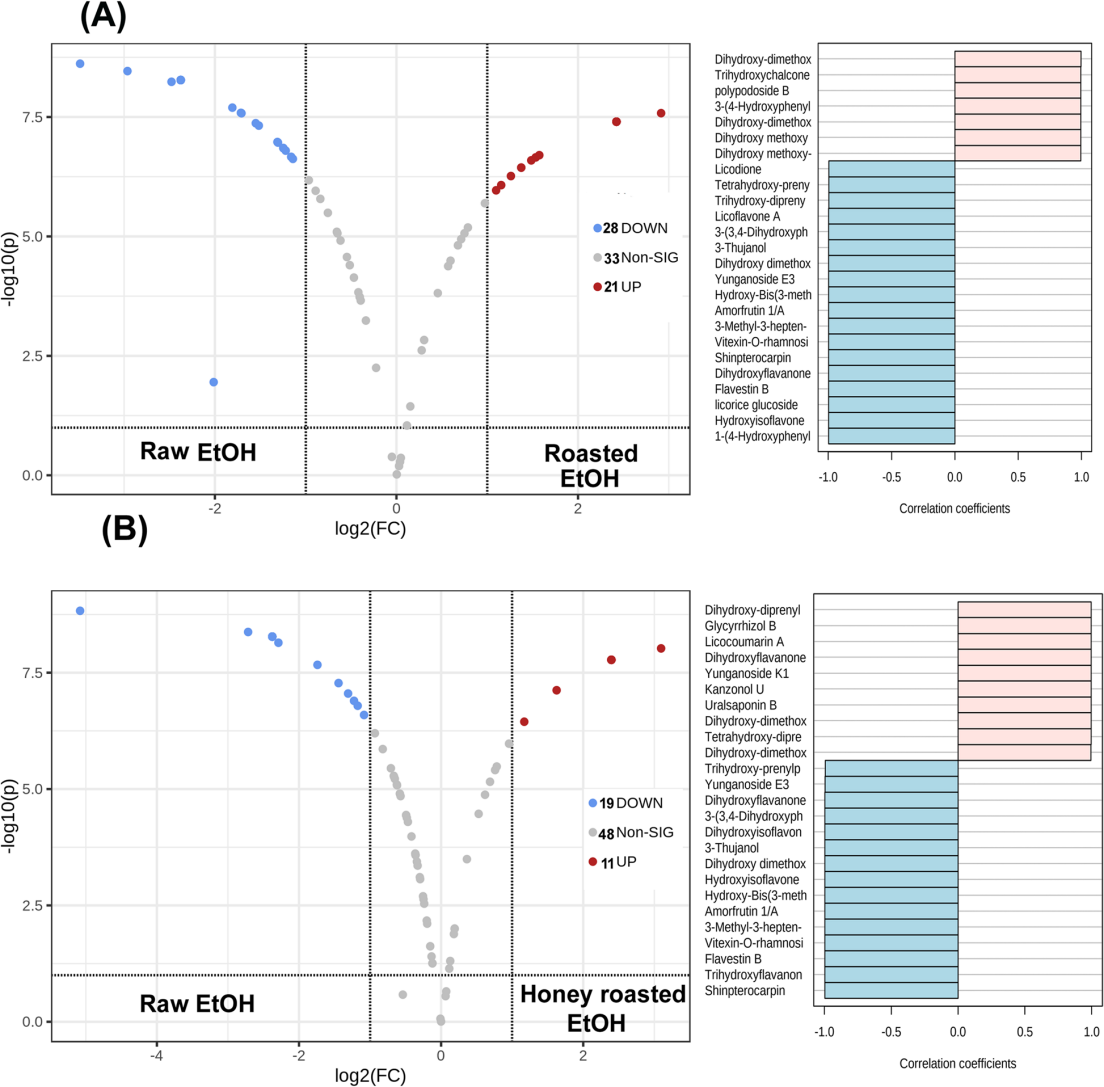
Volcano and coefficient plots of ethanol extracts of raw and roasted samples (**A**), ethanol extracts of raw and honey-roasted samples (**B**)

The up-accumulated and down-accumulated secondary metabolites with roasting and honey-roasting of licorice root samples were visualized using volcano and coefficient plots compared to ethanol and aqueous extracts of raw roots. Figure 4 A shows that after roasting of the licorice samples, 21 metabolites were up-accumulated (red scatter points), 28 were down-accumulated (blue scatter points), and 22 metabolites showed no change when comparing the ethanol extracts of the raw and roasted roots. Dihydroxy-dimethoxy prenylisoflavan, dihydroxy methoxy-prenylisoflavon, dihydroxy-dimethoxyflavone, 3-(4-Hydroxyphenyl)-phenyl-propenone, phenethanamine, methyl-N-vanillyl and licocoumarin A were the main metabolites which showed high accumulation with roasting of the roots samples while hydroxyisoflavone methoxy-O-glucopyranoside, licorice glucoside D1, flavestin B, dihydroxyflavanone-O-rutinoside, shinpterocarpin, vitexin-O-rhamnoside and amorfrutin were the main compounds showing down-accumulation with roasting of the samples.

Meanwhile, honey roasted samples showed an increase in 11 compounds with the decrease in the relative concentrations of 19 compounds while 48 compounds showed no significant changes with honey roasting (Fig. 4B). Dihydroxy-diprenylflavanone, tetrahydroxy-diprenylflavanone, dihydroxy-dimethoxyflavone, dihydroxy-dimethoxy prenylisoflavan, licocoumarin A, glycyrrhizol B were the main metabolites which showed higher accumulation with honey roasting of the roots samples while trihydroxyflavanone-O-pentosyl hexoside, flavestin B, shinpterocarpin, vitexin-O-rhamnoside, hydroxyisoflavone methoxy-O-glycoside were the main compounds showing down-accumulation with honey roasting of the samples.

**Fig. 5:**
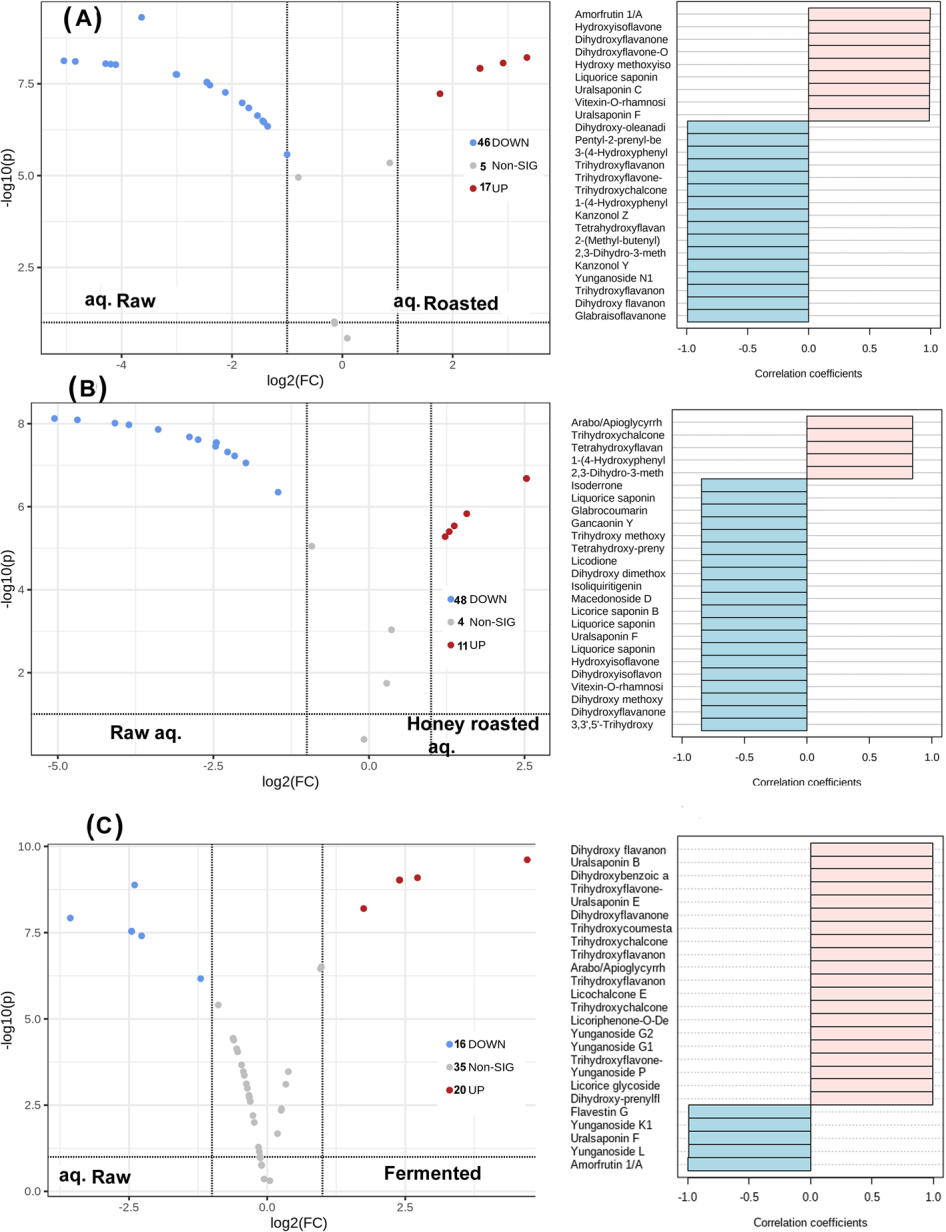
Volcano and coefficient plots of ethanol extracts of aqueous extracts of raw and roasted sample (**A**), aqueous extracts of raw and honey-roasted samples (**B**) and aqueous extracts of raw and fermented licorice roots samples (**C**)

On the other hand, volcano, and coefficient plots of the aqueous extracts of roasted and honey roasted samples compared to raw ones (Fig. 5A and B) depicted a much significant reduction in the accumulation of secondary metabolites where 46 and 48 metabolites were significantly down-accumulated in the roots with roasting and honey-roasting, respectively. Hydroxyisoflavone methoxy-O-glucopyranoside, amorfrutin 1/A, dihydroxyflavanone-O-rutinoside, dihydroxyflavone-O-pentosyl hexoside, vitexin-O-rhamnoside, licorice saponin J2, uralsaponin C and uralsaponin F were among the main secondary metabolites that were significantly reduced with roasting of the roots while 2,3-Dihydro-3-methylfuran, 2-(Methyl-butenyl)-5-(phenylethyl)-benzenediol, and 1-(4-Hydroxyphenyl)-3-(7-methoxybenzofuran-6-yl) propanone glabraisoflavanone A showed significantly higher accumulation in the aqueous extracts of roasted roots. Meanwhile, arabino-glycyrrhizin, tetrahydroxyflavan-O-pentoside, trihydroxychalcone diglycoside, dihydroxy flavanone-O-hexoside, amorfrutin and dihydroxyflavanone-O-rutinoside showed significant down accumulation with honey roasting while 7,8-Dihydro-methylpyrrolopyrimidinone, hydroxy methoxyisoflavone and tetrahydroxy-prenylflavanone displayed significant up-accumulation in the aqueous extracts of honey roasted roots.

Comparing the volcano and coefficient plots of the fermented roots to the raw ones revealed (Fig. 5C) significant increase in the relative concentration of 20 compounds, significant decrease in 16 compounds, where 35 compounds showed no significant change. The oleanane-type triterpene saponins Yunganoside P, Yunganoside G1, Yunganoside G2 as well as the polyhydroxylated derivatives of flavones like trihydroxychalcone diglycoside, trihydroxyflavone-O-Rhamnopyranoside (afzelin), trihydroxyflavanone-O-hexoside, trihydroxyflavanone-O-pentosyl hexoside and trihydroxycoumestan were the main up-accumulated secondary metabolites in fermented roots samples while flavestin G, amorfrutin, dihydrolicoisoflavone A, licoagroisoflavone, glabrene, glabraisoflavanone A and glabridin showed significant down-accumulation with fermentation of licorice roots extracts.

## Discussion

### UPLC-QqQ-MS/MS and chemometric analyses of tested samples

The UPLC-QqQ-MS/MS analysis of raw, roasted, honey roasted and fermented licorice roots samples revealed 133 metabolites belonging to distinct chemical classes; saponins, flavonoids, chalcones, pterocarpans and coumarins being the most abundant groups Supplementary material contains detailed discussion and schematic illustrations of the fragmentation pattern of the key chemicals along with the related literature data (Figures S2-S5).

UPLC-QqQ-MS/MS analysis combined with multivariate statistical analysis was attempted for comprehensive profiling to show similarity and differences between tested samples as well as for assessment the effect of processing procedures on the chemical profile of licorice samples.

The chemical profiles of the studied extracts showed significant differences and these results agreed with previous studies as aqueous extracts scored a high content of triterpene saponins [24] and this content increased by more than 50% upon fermentation of licorice aqueous extract. Roasting results in formation of brown polymers that result from the reaction of sugar and amino acids in the Maillard reaction which are called melanoidins the result behind their presence in roasted and honey-roasted licorice samples [25]. Previous reports showed that roasting affect polar compounds rather than non-polar ones [26]. Moreover, ethanol extracts scored the high percentage of flavonoids and chalcones compared to aqueous extracts [27, 28].

Clustering of samples in HCA heatmap and in SOM indicating that samples were gathered according to the type of extracts while roasting of samples induced greater effect on the chemical profile of polar metabolites rather than non-polar ones leading to the clustering of the aqueous extracts roasted and honey roasted samples away from the aqueous extracts of raw and fermented root samples.

Variable of importance (VIP) plot indicated that the triterpene saponin glycyrrhizin and its hydrolysis product glycyrrhetinic acid, trihdroxychalcone diglycoside, the pyranoisoflavone glabrone, tetrahydroxy prenyl flavanone and the pyranoisoflavan glabridin were the main chemical features responsible for the discrimination of samples where glabrone, tetrahydroxy prenyl flavanone and the pyranoisoflavan glabridin showed significant accumulation in the ethanol extracts of the samples while glycyrrhizin, glycyrrhetinic acid and tetrahydroxy prenyl flavanone accumulated mainly in the aqueous extracts. Glycyrrhetinic acid was found to be particularly enriched in the honey-roasted, fermented, and aqueous extracts of roasted licorice roots.

It can be observed from volcano and coefficient plots that roasting of samples led to the degradation and hydrolysis of glycosides of saponins, isoflavonoids and flavonoids with the appearance of melanoidins and increase in the relative amounts of methoxylated derivatives of isoflavones. Moreover, honey roasting of licorice roots had a less significant effect on the secondary metabolites detected in the ethanol extracts of the roots when compared to roasting without honey. In addition, the process of roasting and honey roasting possessed a more pronounced effect on the relative concentrations and degradation of polar secondary metabolites rather than relatively non-polar ones. Finally, the results obtained indicated that fermentation of licorice roots led to an increase in the relative concentration of phenolic compounds. It has been previously reported that the release of bound phenolic compounds as a result of the destruction of the cell wall structure by microbial enzymes produced during fermentation is primarily responsible for fermentation’s capacity to boost yield and change the profile of phenolic compounds [29].

## Conclusion

In this study, the combination of UPLC-QqQ-MS/MS analysis with multivariate statistical analysis was successfully employed to interrogate the metabolomes of processed (roasted, honey roasted and fermented) and unprocessed licorice samples in addition to track the common alterations in the secondary metabolites comprised in raw licorice by processing. This allowed revealing of the differential metabolites responsible for chemical variations and those chemical variations can be employed as markers for the pre-processing procedures of licorice in addition to scrutinizing processed and unprocessed samples.

### Supplementary Information

**Additional file 1.**

## Data Availability

The datasets used to support this study are available from the corresponding author upon request and after satisfying ethical requirements for their release.

## Abbreviations

ESI: Electrospray ionization
HCA: Hierarchical analysis
OPLS: Orthogonal projection to latent structures
OPLS-DA: Orthogonal projection to latent structures-discriminant analysis
PC: Principal component
QqQ-MS/MS: Triple quadrupole tandem mass spectrometry
RTPCR: Real time polymerase chain reaction
SI: Selectivity index
SOM: Self-organizing map
TQD: Triple quadrupole
UPLC/MS/MS: Ultra performance liquid chromatography-tandem mass
VIP: Variable of importance

## References

[1] Mamedov NA, Egamberdieva D, Ozturk M, Hakeem KR (2019). Phytochemical constituents and Pharmacological effects of Licorice: a Review BT - Plant and Human Health, volume 3: Pharmacology and Therapeutic uses.

[2] Wu S, Wang W, Dou J, Gong L (2021). Research progress on the protective effects of licorice-derived 18β-glycyrrhetinic acid against liver injury. Acta Pharmacol Sin.

[3] Kitagawa I (2002). Licorice root. A natural sweetener and an important ingredient in Chinese medicine. Pure Appl Chem.

[4] Cerulli A, Masullo M, Montoro P, Piacente S. Licorice (Glycyrrhiza glabra, G. Uralensis, and G. inflata) and their constituents as active cosmeceutical ingredients. Cosmetics. 2022;9:1–9. 10.3390/cosmetics9010007.

[5] Bethapudi B, Murugan SK, Nithyanantham M, Singh VK, Agarwal A, Mundkinajeddu D. Chapter 24 - gut health benefits of licorice and its flavonoids as dietary supplements. In: Bagchi D, editor. Ohia Metabolism and Immune Health SEBT-N and FF in BD. editors. Academic Press; 2022. pp. 377–417.

[6] Wu Y, Wang Z, Du Q, Zhu Z, Chen T, Xue Y (2022). Pharmacological effects and underlying mechanisms of licorice-derived flavonoids. Evidence-Based Complement Altern Med.

[7] Mubarik F, Noreen S, Farooq F, Khan M, Khan AU, Pane YS (2021). Medicinal uses of licorice (Glycyrrhiza glabra L.): a Comprehensive Review. Open Access Maced J Med Sci.

[8] Ota M, Xu F, Li Y-L, Shang M-Y, Makino T, Cai S-Q (2018). Comparison of chemical constituents among licorice, roasted licorice, and roasted licorice with honey. J Nat Med.

[9] Li YY, Lee KY, Lee HG (2022). Effects of roasting conditions on Korean rice wine (Makgeolli) with licorice (Glycyrrhiza Uralensis Fischer). Food Sci Biotechnol.

[10] Kong S, Li P, Verpoorte R, Wang J, Zhu C, Dai Y (2022). Synergistic mechanism for the bioactivity fortification of licorice by honey. J Ethnopharmacol.

[11] Bhatt S, Dadwal V, Padwad Y, Gupta M (2022). Study of physicochemical, nutritional, and anticancer activity of Murraya Koenigii extract for its fermented beverage. J Food Process Preserv.

[12] Zhou C, Yang Y, Zhang Z, Liu R, Li W (2011). HPLC with switching wavelength simultaneous determination of seven constituents in licorice and its processed products. Chin J Pharm Anal.

[13] Kuwajima H, Taneda Y, Chen W-Z, Kawanishi T, Hori K, Taniyama T (1999). Variation of chemical constituents in processed licorice roots: quantitative determination of saponin and flavonoid constituents in bark removed and roasted licorice roots. Yakugaku Zasshi J Pharm Soc Japan.

[14] Sung MW, Li PCH (2004). Chemical analysis of raw, dry-roasted, and honey‐roasted licorice by capillary electrophoresis. Electrophoresis.

[15] Su BZ, Zhou Q, Sun LL (2011). Fingerprint of ethyl acetate extract of Glycyrrhizae Radix Et Rhizoma Praeparta. Chin Tradit Pat Med.

[16] Zhou Q, Lv J, Li G, Shi D, Dai Y, Sun L (2010). HPLC fingerprint spectrum of honey-fried Radix Glycyrrhizae. Zhongguo Zhong Yao Za Zhi = Zhongguo Zhongyao Zazhi = China. J Chin Mater Med.

[17] Zhou YZ, Han L, Liu XH, Fu XS, Xu H, Li JS (2012). Study on HPCE fingerprint of Glycyrrhizae Radix et Rhizoma. Chin JMAP.

[18] Li Y, Li Y, Li H, Qi Y, Wu Z, Yang M (2017). Comparative study of microwave-vacuum and vacuum drying on the physicochemical properties and antioxidant capacity of licorice extract powder. Powder Technol.

[19] China Pharmacopoeia Committee. Pharmacopoeia of the People’s Republic of China. 2005.

[20] Muhialdin BJ, Filimonau V, Qasem JM, Ibrahim SA, Algboory HL (2022). Traditional fermented foods and beverages in Iraq and their potential for large-scale commercialization. J Ethn Foods.

[21] Alberti-Dér. Á. LC-ESI-MS/MS methods in profiling of flavonoid glycosides and phenolic acids in traditional medicinal plants: Sempervivum tectorum L. and Corylus avellana L. Budapest: A Ph.D. Thesis, Semmelweis University, Doctoral School of Pharmaceutical Sciences; 2013.

[22] Ghallab DS, Mohyeldin MM, Shawky E, Metwally AM, Ibrahim R, said (2020). Chemical profiling of Egyptian propolis and determination of its xanthine oxidase inhibitory properties using UPLC–MS/MS and chemometrics. LWT- Food Sci Technol.

[23] Kadian N, Raju KSR, Rashid M, Malik MY, Taneja I, Wahajuddin M (2016). Comparative assessment of bioanalytical method validation guidelines for pharmaceutical industry. J Pharm Biomed Anal.

[24] Talib AlSaady A, Al Mousawi H, Saleh R, Omran A, Ghasemian A (2022). Chemical Analysis and Antibacterial Activity of Glycyrrhiza glabra roots. Egypt J Chem.

[25] Park JY, Ji YJ, Seo KH, Lee JY, Kim GS, Kang MH et al. Heat treatment improves Uv photoprotective effects of licorice in human dermal fibroblasts. Processes. 2021;9:1–12. 10.3390/pr9061040.

[26] Bekedam EK. Coffee brew melanoidins. Struct Funct Prop Brown-Colored Coffee Compd. The Netherlands: Ph.D. thesis Wageningen University; 2008. p. 2–15. ISBN: 978–90–8504–951–7.

[27] Wang D, Liang J, Zhang J, Wang Y, Chai X (2020). Natural chalcones in Chinese Materia Medica: Licorice. Evidence-Based Complement Altern Med.

[28] Mohammed SK (2014). Activities of aqueous and ethanolic extracts of Licorice roots. Pakistan J Nutr.

[29] Huynh NT, Van Camp J, Smagghe G, Raes K (2014). Improved release and metabolism of flavonoids by steered fermentation processes: a review. Int J Mol Sci.

