# Supplementary material for: Tracking the effect of roasting and fermentation on the metabolites of licorice root (Glycyrrhiza glabra L.) using UPLC-MS analysis combined with multivariate statistical analysis
Source: BMC Complement Med Ther. 2023 Nov 20;23:419. doi: 10.1186/s12906-023-04239-7 (PMC10662527; doi:10.1186/s12906-023-04239-7)
Supplement: Supplementary file 1 — Additional file 1. [file 12906_2023_4239_MOESM1_ESM.docx]

**Table S1. The codes and methods of samples preparation of different licorice samples.**

| **Licorice sample** | **Code** | **Sample preparation** |
| --- | --- | --- |
| Aqueous extract of raw licorice | AqW1, AqW2, AqW3 | Raw licorice roots are extracted with water without any processing. |
| Fermented licorice | F1, F2, F3 | Samples were prepared mixing 50 g sample with 50 mL water at 20-25 ^◦^C and this mixture was subjected to milling using a mortar and a pestle in order to get a paste. After that, the paste is kept in darkness for 2 days and the soaking this paste in water is done overnight. Finally, the resultant juice (fermented licorice sample (*Erk-sous* beverage)) is obtained through filtration. |
| Aqueous extract of roasted licorice | AqR1, AqR2, AqR3 | Samples are dried in the oven at 180 ◦C till the weight become constant (1 hr) then extracted with water. |
| Aqueous extract of honey-roasted licorice | AqHR1, AqHR2, AqHR3 | Samples were prepared by mixing 50 g of the powdered sample with 12.5 g honey dissolved in 25 mL water followed by sample roasting in oven at 180 ^◦^C for 1 hr then extracted with water. |
| Ethanol extract of raw licorice | EW1, EW2, EW3 | Raw licorice roots are extracted with ethanol without any processing. |
| Ethanol extract of roasted licorice | ER1, ER2, ER3 | Samples are dried in the oven at 180 ◦C till the weight become constant (1 hr) then extracted with ethanol. |
| Ethanol extract of honey-roasted licorice | EHR1, EHR2, EHR3 | Samples were prepared by mixing 50 g of the powdered sample with 12.5 g honey dissolved in 25 mL water followed by sample roasting in oven at 180 ^◦^C for 1 hr then extracted with ethanol. |

**Standard solutions for UPLC-MS quantification**

In order to investigate the variability in the chemical profile of the tested extracts of licorice and to unravel the effect of processing on the quality and quantity of the identified metabolites, all detected metabolites were analyzed in each tested extract based on mean peak area computation using the calibration curves of corresponding standards and the result were readily presented as (mg standard equivalents/g dry extract) [1]. The identified compounds were subjected to semi-quantitative analysis based on their chemical class, using standard compound solutions (1 mg/10 mL) evaluated under the same MS-conditions as previously indicated. Representatives of these chemical classes were quercetin, glycrrhizic acid, esculetin, licochalcone A, ellagic acid, trans-stilbene , and 7, 12-dimethoxy coumestan (Sigma-Aldrich (St. Louis, MO, USA). Standard solutions were prepared by weighing accurate weight of each standard (10 mg). Each standard was separately placed in 10-mL volumetric flask and HPLC-grade methanol was added. After that, the solution was serially diluted to the working concentrations over the reliable range 0.0125 – 0.75 mg/mL using the same solvent. Five μL aliquots of each standard compound were injected onto the chromatographic system in duplicates for different concentration levels. The standard calibration curves were established by plotting peak areas of the standards as the analytical responses against their known concentration concentrations. Validation parameters like linearity, limit of detection (LOD) and limit of quantiﬁcation (LOQ) were assessed based on FDA guideline on bioanalytical method validation [2] To assure data quality, the precision and reproducibility of the data were assessed using the standard mixing solution and QC samples. Over the course of 5 days, the standard mixed solution was continually injected 10 times (5 times each in positive and negative ion modes) (5 times each in positive and negative ion modes). (**Table S2**).

**Standard solutions used for confirming the identification of metabolites.**

Amorfrutin 1/A, Dihydroxyflavanone-O-rutinoside, Trihydroxyflavone-O-Rhamnopyranoside (afzelin), Isoliquiritin apioside, Vitexin-O-rhamnoside, Uralsaponin E, Trihydroxyflavone-glucoside, licorice glucoside D1, Uralsaponin F, Liquorice saponin J2, Yunganoside G2, Licorice saponin G2) 24-Hydroxyglycyrrhizin, Yunganoside P, Flavestin B, Isoliquiritigenin, Glycyrrhizin, Licodione, Gancaonin Y, Glabrocoumarin, Uralsaponin C, Licochalcone E, Phaseol, kanzonol A, Glycyrrhizol B, Gancaonin W, Licoisoflavone B, Licoagrodione, Licoagroisoflavone, Glabridin, Glabrone, Licocoumarin A, Kanzonol Z, Glabrene, and Shinpterocarpin standards were used for confirming the identity of metabolites. These standards were previously isolated and supplied by the Department of Pharmacognosy, Faculty of Pharmacy, Alexandria University. Structural determination of the isolated standards was established by different spectral analyses (UV, MS, NMR, and 2D-NMR). The purity of the standards was more than 95 % as checked by HPLC.

**Table S2: Linearity and sensitivity parameters for quercetin, glycrrhizic acid, esculetin, licochalcone A and ellagic acid**

| **Compound** | **Linearity range (mg /mL)** | **Slope (a)** | **Intercept (b)** | **Corelation coefficient (r)** | **LOD (mg /mL)** | **LOQ (mg /mL)** |
| --- | --- | --- | --- | --- | --- | --- |
| **Quercetin** | **0.013-0.255** | 1.55*10^7^ | -6.55*10^3^ | **0.996** | **0.007** | **0.032** |
| **Quercetrin** | **0.011-0.201** | 1.67*10^7^ | -7.45*10^4^ | **0.997** | **0.008** | **0.022** |
| **glycyrrhizic acid** | **0.021-0.522** | 2.25*10^7^ | -8.20*10^4^ | **0.994** | **0.011** | **0.040** |
| **Esculetin** | **0.010-0.6** | 4.55*10^7^ | -7.20*10^4^ | **0.997** | **0.008** | **0.030** |
| **Licochalcone A** | **0.04-0.7** | 1.65*10^7^ | -9.91*10^4^ | **0.998** | **0.013** | **0.050** |
| **Ellagic acid** | **0.05-0.8** | 1.71*10^7^ | -8.89*10^4^ | **0.994** | **0.015** | **0.060** |
| ***Trans*-stilbene** | **0.04-0.9** | 1.42*10^7^ | -7.00*10^4^ | **0.992** | **0.012** | **0.080** |
| **7,12-Dimethoxy-coumestan** | **0.013-0.238** | 2.10*10^7^ | -6.36*10^3^ | **0.994** | **0.009** | **0.071** |
|  |  |  |  |  |  |  |

**Experimental conditions as in Section 2.3.2. For each calibration curve the equation is y= ax + b, where y is the peak area, x is the concentration of the standard (mg/mL), a is the slope, b is the intercept, r the correlation coefﬁcient, LOD is the limit of detection and LOQ is the limit of quantitation.**

**Table S3: UPLC-MS metabolite profiling data from all *glycrrhiza glabra* samples representing the content of each variable (peak areas) calculated as (expressed as mg standard equivalents/g dry extract)**

| **Compounds** | **Chemical class** | **Ethanol extact raw** | **Ethanol extract Roasted** | **Fermented** | **Aqueous extract Honey roasted** | **Ethanol extract Honey roasted** | **Aqueous extract Raw** | **Aqueous extract**  **Roasted** |
| --- | --- | --- | --- | --- | --- | --- | --- | --- |
| **7,8-Dihydro-methylpyrrolopyrimidinone*** | **pyrimidine** | **0.4512141** | **0.282861** | **1.08E+00** | **0.572062022** | **8.49E-01** | **8.36E-01** | **9.56E-01** |
| **Dihydromethylfuran ^#^** | **Dihydrofuran** | **0** | **0.035026** | **0** | **0** | **0.0575931** | **0.048757** | **0** |
| **2-(Methyl-butenyl)-5-(phenylethyl)-benzenediol ^#^** | **Resorcinols** | **0** | **0** | **0** | **0** | **0** | **0.0275436** | **0** |
| **Pentyl-2-prenyl-benzenediol-3-Me ether ^#^** | **Resorcinols** | **0** | **0** | **0** | **0** | **0** | **0.0374859** | **0** |
| **Dihydroxybenzoic acid-Xylopyranosyl ester ^#^** | **Phenolic acid** | **0** | **0** | **0.0096871** | **0** | **0** | **0** | **0** |
| **Hydroxy-Bis(3-methyl-2-butenyl)-5-(2-phenylethenyl)-benzenediol******* | **Chalcone** | **0.0211613** | **0** | **0** | **0** | **0** | **0** | **0** |
| **Hydroxyphenyl)-3-(7-methoxybenzofuran-6-yl) propanone ^#^** | **Ketone** | **0.5912642** | **0.056936** | **0** | **0.238436306** | **0.178647** | **7.31E-01** | **0** |
| **3-Methyl-heptenone ^#^** | **Ketone** | **6.97E-01** | **0** | **0** | **0** | **0** | **0** | **0** |
| **3-(4-Hydroxyphenyl)-phenyl-propenone******* | **Chalcone** | **0** | **0.139703** | **0** | **0** | **0** | **0.0455099** | **0** |
| **3-Thujanol***** | **Monoterpene** | **0.099079** | **0** | **0** | **0** | **0** | **0** | **0** |
| **3,3',5'-Trihydroxy-4-methoxybibenzyl^##^** | **stilbene** | **0** | **0** | **0** | **0** | **0** | **0** | **0.02074091** |
| **Dihydroxyflavanone-Di-O-hexoside**** | **Flavanone glycosides** | **0.1629208** | **0.123581** | **0** | **0.100186641** | **0** | **0** | **0** |
| **Amorfrutin #** | **phenolic acid** | **0.1805651** | **0** | **0** | **0.206340273** | **0** | **0.1684457** | **0.01843653** |
| **Dihydroxyflavanone-O-Hydroxypropanoyl hexoside**** | **Flavanone glycosides** | **0** | **0.016543** | **0** | **0** | **0** | **0** | **0.03170296** |
| **3-(3,4-Dihydroxyphenyl)-2-propenoic acid- docosyl ester ^#^** | **Phenolic acid ester** | **0.304941** | **0** | **0** | **0** | **0** | **0** | **0** |
| **Tetrahydroxyflavan-O-pentoside**** | **flavane glycosides** | **0.0341174** | **0.091649** | **0** | **0.025608472** | **0.0417374** | **0.0985717** | **0** |
| **Dihydroxy dimethoxy-O-hexoside**** | **Isoflavone glycosides** | **0.0696452** | **0** | **0** | **0** | **0** | **0** | **0** |
| **Trihydroxychalcone-O-[Rhamnopyranosyl-glucopyranoside] ******* | **Chalcone glycosides** | **0.2581685** | **0.140354** | **0.2344728** | **0.146707123** | **0.0637154** | **0.2020064** | **0** |
| **Dihydroxyflavanone-O-[rutinoseide]**** | **Flavanone glycosides** | **0.0809814** | **0** | **0.5632205** | **0.019896258** | **0.0297965** | **0.0278256** | **6.99E-01** |
| **Dihydroxyflavone-O-[pentosyl hexoside]**** | **Flavone glycosides** | **0.1016351** | **0.047991** | **0.2009862** | **0.066671706** | **0.0363937** | **0.0125534** | **0.16882012** |
| **Trihydroxyflavone-O-Rhamnopyranoside (afzelin)**** | **Flavone glycosides** | **0.0821247** | **0.155169** | **0.3134839** | **0.055652772** | **0** | **0.0905414** | **0** |
| **Dihydroxyflavanone-O-pentosyl hexoside**** | **Flavanone glycosides** | **0** | **0** | **0.0387765** | **0** | **0** | **0** | **0** |
| **Isoliquiritin apioside******* | **Chalcone glycosides** | **1.77E+00** | **0.59946** | **2.31E+00** | **7.75E-01** | **6.18E-01** | **1.08E+00** | **1.24E+00** |
| **Hydroxyflavanone-Me ether-O-hexoside**** | **Flavanone glycosides** | **0** | **0** | **0.0574092** | **0** | **0** | **0** | **0.05123283** |
| **Vitexin-O-rhamnoside**** | **Flavone glycosides** | **0.1205012** | **0** | **0.3465624** | **0** | **0** | **0.0427083** | **0.322116** |
| **Dihydroxy flavanone-O-hexoside**** | **Flavanone glycosides** | **0.1154331** | **0.267137** | **7.17E-01** | **0.122932069** | **0.0744409** | **0.2137523** | **0.03330433** |
| **Yunganoside G1***** | **Sapononis(oleane-type triterpene)** | **0** | **0** | **0** | **0.12806552** | **0** | **0** | **0** |
| **Trihydroxyflavanone-O-[pentosyl-hexoside]**** | **Flavanone glycosides** | **0.0979752** | **0.041846** | **0.2569868** | **0.018492984** | **0** | **0.0628202** | **0** |
| **Trihydroxyflavanone-O-hexoside **** | **Flavanone glycosides** | **0** | **0** | **0.047795** | **0** | **0** | **0.0162986** | **0** |
| **Trihydroxychalcone-O-[Apiofuranosyl-glucopyranoside]******* | **Chalcone glycosides** | **8.48E-01** | **1.79E+00** | **0** | **6.81E-01** | **0** | **0** | **0** |
| **Uralsaponin E***** | **Oleana type triterpenes** | **0** | **0** | **0.010884** | **0** | **0** | **0** | **0** |
| **Trihydroxyflavone-O-Glucuronopyranoside**** | **Flavone glycosides** | **0** | **0** | **0.0094698** | **0** | **0** | **0** | **0** |
| **dihydroxyisoflavone-dimethoxy-O-hydroxyphenyl propanoyl-hexoside**** | **Isoflavone glycosides** | **0.0174292** | **0.009858** | **0.0710707** | **0** | **0** | **0** | **0.09237308** |
| **licorice glucoside D1**** | **Flavanone glycosides** | **0.4174352** | **0** | **0** | **0.196482354** | **0** | **0** | **0** |
| **Trihydroxychalcone-O-Hydroxycinnamoyl-apiofuranosyl-glucopyranoside******* | **Chalcone** | **0.2814361** | **0.145349** | **0.2430072** | **0.122135543** | **0** | **0** | **0** |
| **Hydroxyisoflavone methoxy-O-glucopyranoside**** | **Isoflavone glycosides** | **0.2169476** | **0.031382** | **0.5027449** | **0** | **0** | **0.0269202** | **7.75E-01** |
| **Licorice glycoside C1**** | **Flavanone glycosides** | **0** | **0** | **0.0139528** | **0** | **0** | **0** | **0** |
| **yunganosides L 1 or J1***** | **Sapononin(oleane-type triterpene)** | **0.1083829** | **0** | **0** | **0.057274172** | **0** | **0** | **0** |
| **Liquorice saponin F3***** | **Sapononin(oleane-type triterpene)** | **0** | **0** | **0** | **0** | **0** | **0** | **0.00923518** |
| **Uralsaponin F***** | **Sapononin(oleane-type triterpene)** | **0** | **0** | **0** | **0** | **0** | **0** | **0.00826608** |
| **Liquorice saponin J2***** | **Triterpene saponin** | **0** | **0** | **0.0643786** | **0** | **0** | **0.0119129** | **0.15157853** |
| **Tetrahydroxychalcone-Me ether******* | **Chalcone-ether** | **0.1847619** | **0.302789** | **0.0719774** | **0.309310737** | **0.1232011** | **0.0994931** | **0.05947712** |
| **Trihydroxychalcone-O-Hydroxy-methoxycinnamoyl-apiofuranosyl-glucopyranoside******* | **chalcone-glycoside** | **0** | **0.179406** | **0** | **0** | **0** | **0** | **0** |
| **Yunganoside K1***** | **Triterpene saponin** | **0** | **0.078304** | **0** | **0.055138172** | **0.0081641** | **0** | **0.0302311** |
| **Yunganoside K2***** | **Saponin glycoside** | **0** | **0.06065** | **0** | **0** | **0** | **0** | **0** |
| **Yunganoside K3***** | **Triterpene saponin** | **0** | **0** | **lower than LOD** | **0** | **0** | **0** | **0** |
| **Dihydroxy-dimethoxyflavone*** | **Flavone** | **0** | **0.070151** | **0** | **0.104441597** | **0** | **0** | **0** |
| **Uralsaponin M***** | **Triterpene Saponin** | **0** | **0** | **4.99E-01** | **0** | **0.0911724** | **0.211029** | **6.29E-01** |
| **(licorice saponin G2) 24-Hydroxyglycyrrhizin***** | **Triterpene Saponin** | **0** | **0.054261** | **0** | **0** | **0** | **0** | **0** |
| **Yunganoside P***** | **Triterpene Saponin** | **0** | **0** | **0.0113059** | **0** | **0** | **0** | **0** |
| **Dihydroxyflavanone-O-Indolylcarbonyl-pentosyl hexoside**** | **Flavanone glycoside** | **0** | **0.158287** | **0** | **0.093608589** | **0** | **0** | **0** |
| **Yunganoside N1***** | **Triterpene Saponin** | **0** | **0** | **0** | **0** | **0** | **0.021256** | **0** |
| **Licorice saponin B2 or Isomer of licorice saponin B2***** | **Triterpene Saponin** | **0** | **0** | **0** | **0** | **0** | **0** | **0.00736985** |
| **Dihydroxy-methoxy isoflavan*** | **Isoflavan** | **0** | **0.020771** | **0** | **0** | **0** | **0** | **0** |
| **Dihydroxy- methoxy prenylisoflavone*** | **Isoflavone** | **0** | **0.018146** | **0** | **0** | **0** | **0** | **0** |
| **Licoricesaponin K2***** | **Triterpene Saponin** | **0** | **0** | **0.0638569** | **0** | **0.0182234** | **0.0420764** | **0.07301396** |
| **Macedonoside D***** | **Triterpene Saponin** | **0** | **0** | **0.0633754** | **0** | **0** | **0** | **0.07144843** |
| **Flavestin B ^#^** | **Prenylated resorcinol** | **0.0349684** | **0** | **0** | **0** | **0** | **0** | **0** |
| **Macedonoside B***** | **Triterpene Saponin** | **0** | **0** | **0.1310758** | **0** | **0.0117973** | **0.0565248** | **0.15083102** |
| **Uralsaponin B***** | **Triterpene Saponin** | **0** | **0** | **0.3919625** | **0.037568569** | **0.0747866** | **0** | **0.0658922** |
| **Isoliquiritigenin******* | **Chalcone** | **0.0923465** | **0.09149** | **0.0867287** | **0.066938203** | **0** | **0** | **0.4182309** |
| **Glycyrrhizic acid (Glycyrrhizin)***** | **Triterpene glycoside** | **0.2106696** | **0.312615** | **4.4721676** | **0.14885762** | **1.2459818** | **2.5118861** | **5.22027544** |
| **Trihydroxycoumestan-Me ether^###^** | **Coumestan** | **0** | **0** | **1.0834826** | **0** | **0** | **0** | **0** |
| **Dihydroxy-dimethoxy isoflavone*** | **Isoflavone** | **0** | **0** | **0.4579544** | **0** | **0** | **0** | **0.24809916** |
| **Hydroxy-methoxy isoflavone *** | **Isoflavone** | **0.3020932** | **0.408988** | **0.1888757** | **0.252698564** | **0.1046611** | **0.0164588** | **0.30601319** |
| **Licodione******* | **dihydrochalcones** | **0.0437619** | **0.017427** | **0.1757835** | **0.030228967** | **0** | **0** | **0.20609986** |
| **Dihydroxy-oleanadienoic acid-O-Glucuronopyranosyl-rhamnopyranoside***** | **Triterpene Saponin** | **0** | **0** | **0** | **0** | **0** | **0.0153801** | **0** |
| **Xambioona*** | **flavanone** | **0** | **0** | **0.0901737** | **0** | **0.3276147** | **0** | **0.11775288** |
| **Trihydroxy-oleanadienoic acid-O-Glucuronosyl-rhamnoside***** | **Triterpene Saponin** | **0** | **0** | **0.2102706** | **0** | **0.011368** | **0.0641508** | **0.27693141** |
| **Glycyrrhizic acid isomer (Glycyrrhizin isomer) ***** | **Triterpene glycoside** | **0.0538782** | **0.041145** | **2.0878832** | **0.034974813** | **0.3037811** | **0.8931624** | **2.36593444** |
| **Tetrahydroxy-prenylchalcone******* | **Chalcone** | **0.6015796** | **0.185393** | **0.0641152** | **0.37984765** | **0** | **0.034217** | **0.08574831** |
| **trihydroxy-methoxyprenylisoflavone*** | **prenyl Isoflavone** | **0** | **0** | **0.0525461** | **0** | **0** | **0** | **0.08116194** |
| **Trihydroxy-prenylflavanone*** | **Flavanone** | **0.1238557** | **0.092472** | **0** | **0.106665694** | **0** | **0** | **0** |
| **Gancaonin Y*** | **Isoflavan** | **0** | **0** | **0.1842564** | **0** | **0** | **0** | **0.25936926** |
| **Glabrocoumarin****** | **Coumarin** | **0** | **0** | **0.1566243** | **0** | **0** | **0.0428005** | **2.28E-01** |
| **Gancaonin V***** | **Dihydrophenanthrene** | **0.0527582** | **0.037781** | **0** | **0.027471623** | **0** | **0** | **0** |
| **Arabo/Apioglycyrrhizin***** | **Triterpene glycoside** | **0** | **0** | **lower than LOD** | **0** | **0.0230445** | **0** | **0** |
| **Uralsaponin C***** | **Triterpene glycoside** | **0** | **0** | **0.1982298** | **0** | **0.021606** | **0.0366948** | **0.2810378** |
| **Tetrahydroxy-prenylflavanone*** | **Flavanone** | **0** | **0** | **1.27E+00** | **0** | **0.1513686** | **0.4610227** | **1.68E+00** |
| **Chiricanin B****** | **Coumarin** | **0.1319345** | **0.083661** | **0** | **0.119657422** | **0** | **0** | **0** |
| **Liquorice saponin C2***** | **saponin (oleanane type triterpene)** | **0** | **0** | **0.1372115** | **0** | **0** | **0** | **0.15035509** |
| **Isoderrone *** | **Pyranoisoflavone** | **0.0719174** | **0.030739** | **0.1734722** | **0.080277506** | **0** | **0** | **0.2348901** |
| **Licochalcone E******* | **chalcone** | **0** | **0** | **0.0503417** | **0** | **0** | **0** | **0** |
| **Tetrahydroxy-diprenylflavanone*** | **flavanone** | **0** | **0** | **0** | **0.016673466** | **0** | **0** | **0** |
| **Trihydroxy-methoxy isoflavone*** | **isoflavone** | **0** | **0** | **0.131578** | **0** | **0** | **0** | **0.1746141** |
| **yunganoside I2***** | **saponin (oleanane type triterpene)** | **0** | **0** | **0.2820751** | **0** | **0.0547834** | **0.0914071** | **0.32577446** |
| **Trihydroxy-prenylchalcone-Me ether******* | **chalcone** | **0.1838553** | **0.461558** | **0** | **0.19769755** | **0** | **0** | **0** |
| **Dihydroxy-prenylflavanone*** | **flavanone** | **0.408293** | **0.282549** | **8.29E-01** | **0.271286467** | **0.5991428** | **8.57E-01** | **0.26149999** |
| **Phaseo^l###^** | **coumestan** | **2.5457816** | **0.411865** | **0** | **0.211561567** | **0** | **0** | **0** |
| **Kanzonol A ******* | **chalcone** | **0.3013941** | **0.307803** | **0.1785058** | **0.26796181** | **0** | **0** | **0.1823626** |
| **Glycyrrhizol B*** | **pterocarpene** | **0** | **0** | **0** | **0.01103108** | **0** | **0** | **0** |
| **Yunganoside L***** | **saponin (oleanane type triterpene)** | **0** | **0** | **0.0425629** | **0** | **0** | **0.1482113** | **4.90E-01** |
| **Gancaonin W****** | **Coumarin** | **0.026277** | **0.028702** | **0.0188022** | **0.036697788** | **0** | **0** | **0.02945191** |
| **Trihydroxy-prenylstilbene^##^** | **stilbene** | **0.0371626** | **0.02274** | **0** | **0.03650048** | **0** | **0** | **0** |
| **Licoisoflavone B*** | **Pyranoisoflavone** | **0.0876894** | **0.128326** | **0** | **0.086425634** | **0.055506** | **0** | **0** |
| **Kanzonol U*** | **2-arylbenzofuran flavonoids** | **0** | **0** | **0** | **0.134089993** | **0** | **0** | **0** |
| **Flavestin G^##^** | **stilbene** | **0.8361632** | **0.351741** | **0** | **0.656872465** | **0** | **0** | **0.05027548** |
| **Licoagrodione^##^** | **stilbene** | **0.0926194** | **0.088576** | **0** | **0.073186828** | **0** | **0** | **0** |
| **Licoagroisoflavone*** | **isoflavone** | **0.6494614** | **0.69492** | **0** | **0.455238864** | **0** | **0** | **0.03146958** |
| **Trihydroxy-prenylpterocarpan-Me ether*** | **pterocarpan** | **0.1377387** | **0.088904** | **0** | **0.050206313** | **0** | **0.420802** | **0.41059763** |
| **Trihydroxy-prenylpterocarpan-Didehydro-Me ether*** | **pterocarpan** | **0.0379905** | **0.038275** | **0** | **0.023764261** | **0** | **0** | **0** |
| **Glycyrrhizaisoflavone C*** | **isoflavone** | **0.0299355** | **0.082017** | **0** | **0.065918788** | **0** | **0** | **0** |
| **Glycyrrhizaflavonol A*** | **Pyranoflavonol** | **0.4362156** | **0.150921** | **0.0168945** | **0.334306234** | **0** | **0** | **0.02564128** |
| **Glabridin*** | **Pyranoisoflavan** | **0.4845921** | **0.764992** | **0.0171291** | **0.733235856** | **0** | **0** | **0.03303602** |
| **Glabrone*** | **Pyranoisoflavone** | **0.7641318** | **0.775804** | **0.0276937** | **0.686493401** | **0** | **0** | **0.04426728** |
| **Hydroxy-prenylflavanone*** | **flavanone** | **2.60E-01** | **0.176346** | **0** | **0.20020248** | **0** | **0** | **0** |
| **Dihydroxy-diprenylflavanone*** | **flavanone** | **0.0554686** | **0.121377** | **0** | **0.464544701** | **0** | **0** | **0** |
| **Glyasperin G^###^** | **coumestan** | **0.0794368** | **0.320024** | **0** | **0.893260355** | **0** | **0** | **0** |
| **Cyclolicocoumarone**** | **arylbenzofuran** | **0.0242731** | **0.053998** | **0** | **0.041647565** | **0** | **0** | **0** |
| **Licoflavone A*** | **flavone** | **0.1278305** | **0.023015** | **0** | **0.098314976** | **6.71E-01** | **0** | **0** |
| **Dihydroxy-oxo-12-oleanen-oic acid lactone***** | **triterpene (Oleane type)** | **5.63E-01** | **0.251032** | **0** | **0.416837987** | **0** | **0** | **0** |
| **Glabraisoflavanone A *** | **isoflavanone** | **0.8884358** | **0.656269** | **0** | **0.915130863** | **0** | **0.215387** | **0.02239649** |
| **Licocoumarin A****** | **coumarin** | **0** | **0.055513** | **0** | **0.01082965** | **0** | **0** | **0** |
| **Kanzonol Z*** | **Flavanone** | **0** | **0** | **0** | **0** | **0** | **0.0878739** | **0** |
| **Kanzonol Y******* | **chalcone** | **0.2779445** | **0.218703** | **0** | **0.25236194** | **0** | **0.0778169** | **0** |
| **Licochalcone A******* | **chalcone** | **0.1623227** | **0.196755** | **0** | **0.168046647** | **0** | **0** | **0** |
| **Dihydrolicoisoflavone A*** | **isoflavanone** | **0** | **0** | **0** | **0** | **0** | **0** | **0.13446349** |
| **Kanzonol B******* | **chalcone** | **0.0668839** | **0.078907** | **0** | **0.054930163** | **0** | **0** | **0** |
| **Dihydroxy-oleanadienoic acid-O-Glucuronopyranoside***** | **saponin (oleanane type triterpene)** | **0.121028** | **0.041651** | **0** | **0.233717448** | **0** | **0** | **0.04388818** |
| **Glabrene *** | **Pyranoisoflavene** | **0.087628** | **0.149073** | **0** | **0.098527429** | **0** | **0** | **0.05863685** |
| **Yunganoside E3***** | **saponin (oleanane type triterpene)** | **0.0901062** | **0** | **0** | **0.029165388** | **0** | **0** | **0** |
| **Shinpterocarpin*** | **pterocarpan** | **0.2085388** | **0** | **0** | **0.006486086** | **0** | **0** | **0** |
| **Hispaglabridin B*** | **Pyranoisoflavan** | **0.0387739** | **0.110115** | **0** | **0.064464836** | **0** | **0** | **0** |
| **Trihydroxy-diprenylisoflavan*** | **isoflavan** | **0.1094025** | **0.031179** | **0** | **0.090529056** | **0** | **0** | **0** |
| **Glyinflanin A******* | **chalcone** | **0.5014661** | **0.295891** | **0** | **0.430654267** | **0** | **0** | **0** |
| **Erypoegin B, O-De-Me*** | **Pyranoisoflavene** | **0.0740083** | **0.062644** | **0** | **0.117367636** | **0** | **0** | **0** |
| **Dihydroxy-dimethoxy prenylisoflavan*** | **isoflavan** | **0.0902503** | **0.669694** | **0** | **0.273798388** | **0** | **0** | **0** |

**Data are expressed as the average of three determinations (n=3).**

*** Compounds are expressed** **as quercetin acid equivalent**

**** Compounds are expressed as quercetrin acid equivalent**

******* **Compounds are expressed as glycrrhizic acid** **equivalent**

****** Compounds are expressed as esculetin** **equivalent**

********* **Compounds are expressed as licochalcone A equivalent**

**# Compounds are expressed ellagic acid equivalent**

**## Compounds are expressed as *Trans*-stilbene equivalent**

**### Compounds are expressed as 7,12-dimethoxy coumestan equivalent**

**3.1.1. Flavonoids**

Fifty one peaks represented this class, among which 19 peaks were identified as flavanones, 2 peaks represented isoflavanones, 7 peaks were flavones, 1 peak represented flavone C-glycosides, 13 peaks represented isoflavones, 2 peaks were identified as isoflavenes, 1 peak represented flavanes in addition to 6 peaks were identified as isoflavanes.

**3.1.1.1 Flavanones**

Fifty-one peaks represented this class, among which 19 peaks (**peaks 17, 19, 24, 27, 29, 31, 33, 34, 39, 42, 56, 74, 79, 85, 90, 94, 112, 113 and 120)** were flavanones. Flavanone glycosides were represented in the tested extracts by 11 peaks; **17, 19, 24, 27, 29, 31, 33, 34, 39, 42** and **56**. The daughter fragment (M-H-162) that indicated the loss of a hexose unit was detected as a characteristic fragment of compounds **31, 34, 39** and **42**. Meanwhile, **peaks 27, 33** and **56** showed a mass fragment (M-H-294) at 255 Da, 271 Da and 400, respectively indicating a loss of a pentose and hexose sugar units that linked together at the same position of the aglycone. On the other hand, **peak 24** was rutinoside as it showed a characteristic daughter peak (M-H-308). Moreover, **peak 17** showed two daughter fragments at 419 Da and 257 Da indicating that the two hexose units attached to two different positions of the aglycone. Furthermore, **peaks 19** showed a daughter fragment at 257 Da (M-H-232) that result from the loss of hydroxypropanoyl- hexose. Peaks **74, 79, 85, 90, 94, 112, 113** and **120** were identified as flavanone aglycones. The characteristic fragments of flavanones are due to loss of water, CO and CO_2_[5, 6] in addition to loss of a prenyl group (loss of 69 Da)[7] as in prenylated flavanones (**Peaks 79, 85, 94** and **112**) and loss of two prenyl groups (loss of 138 Da) as in diprenylated flavanones (**Peaks 90** and **113**).

**3.1.1.2. Isoflavanones**

Two peaks represented this class **(Peaks 117 and 123)**. Regarding **peak 117**, it showed its quasimolecular ion (M-H) at 391 Da along with its characteristic daughter peaks that result from loss of CO, CO_2_, water and prenyl group in addition to RDA rearrangement fragment A^1,3^ thar detected at 204 Da indicating the substitution of ring A with a hydroxyl group and an isoprenyl group. On the other hand, **peak 123** showed its quasimolecular ion (M-H) at 355 Da along with its characteristic MS^2^ fragments that were due to loss of water, CO, CO_2_ and prenyl group in addition to RDA fragment A^1,3^ at 151 Da indicating the presence of 2 hydroxyl groups in ring A. Meanwhile RDA fragment B^1,3^ at 202 Da showing that the substituents of ring B are 2 hydroxyl group and one prenyl group. By referring to literature, **peaks 117 and 123** were tentatively identified as glabraisoflavanone A and dihydrolicoisoflavone A, respectively (Fukai et al. 2002;Suman et al. 2009;Montoro et al. 2011; Zhang et al. 2018;Fabre et al. 2001; Xu et al. 2013; Lim 2015)

**3.1.1.3.** **Flavones**

Five peaks (**peaks 25, 26, 37, 52** and **116**) represented this chemical class in addition to one peak (**peak 30**) represented flavone C-glycosides. **Peaks 25**, **26** and **37** were O-glycosides. **Peak 25** was a pentosyl hexoside as it showed a characteristic daughter peak (M-H-294). Meanwhile**, peak 26** was a rhamnoside due to the presence of a fragment (M-H-146) [7] and **peak 37** showed a daughter fragment at (M-H-176) indicating a loss of one glucuronic acid unit [7]. **Peak 30** showed a characteristic daughter fragment at 431 Da that result from rhamnose unit loss indicating that the aglycone part to be vitexin. Moreover, there were two characteristic fragments that result from sugar part fragmentation at 311 and 341 Da and compound 30 was identified as vitexin-O-rhamnoside [14]. On the other hand, **peaks 52** and **116** were aglycones, the most important daughter fragments were due to loss of water, CO and CO_2_. **Peak 52** showed RDA arrangement fragments at A^1,3^ at 153 Da indicating the presence of two hydroxyl groups in ring A and B^1,3^ at 166 indicating the presence of two methoxyl groups in ring B [7, 12]. On the other hand, **peak 116** was a prenylated flavone that show a fragment (M-H-69) due to loss of a prenyl group [7] in addition to two RDA rearrangement A^1,3^ at 204 Da indicating the Prescence of a prenyl group and a hydroxy group in ring A and B^1,3^ at 120 Da as ring B contains one hydroxyl group [7]. **Peak 102** represented 2-arylbenzofuran flavonoid that showed its quasi-molecular ion peak (M-H) at 307 Da along with its characteristic daughter peaks that result from water and CO_2_ loss in addition to the diagnostic cleavage of C4H8O that detected at 72 Da [15]. **Peak 109** was a flavonol that showed a quasimolecular ion peak (M-H) at 369 Da and it showed characteristic MS^2^ fragments due to loss of water, CO, CO_2_ an CH_3_. By referring to literature, **peaks 25, 26, 30, 37, 52, 102, 109** and **116** were tentatively identified as dihydroxyflavone-O-pentosyl hexoside, afzelin, vitexin-O-rhamnoside, trihydroxyflavone-O-glucuronopyranoside, dihydroxy-dimethoxyflavone, kanzonol U, glycyrrhizaflavonol A and licoflavone A, respectively (Kovács et al. 2008;Montoro et al. 2011; Zhang et al. 2018;Fabre et al. 2001; Lim 2015; Ghallab et al. 2021).

**3.1.1.4. Isoflavones**

This class was represented by 13 peaks (**22, 38, 41, 60, 69, 70, 78, 88, 91, 101, 105, 108 and 111**), among which peaks **22, 35 and 41** were isoflavone glycosides and they showed their characteristic daughter ions that result from the loss of sugar units. Regarding **peaks 22 and 41**, they showed the fragments (M+H-162) and (M-H-162), respectively. These fragments indicated the loss of one hexose unit from each isoflavone [7]. On the other hand, **peak 38** showed a MS^2^ fragment at 309 Da indicating the loss of hydroxy phenyl propanoyl hexose unit. Meanwhile, peaks **(60, 69, 70, 78, 88, 91, 101, 105, 108 and 111**) were isoflavone aglycones. **Peak 78** was a prenylated isoflavone and it showed a fragment at 300 Da that result from the loss of a prenyl group. Moreover, **peaks 88, 101** and **111** were pyranoisoflavones [17, 18]. **Peak 88** showed RDA rearrangement fragments A^1,3^ at 153 Da indicating the presence of two hydroxyl groups in ring A and B^1,3^ at 201 indicating the presence of a pyrano group attached to ring B. Meanwhile, **Peak 101** A^1,3^ fragment was at 151 Da indicating the presence of two hydroxyl groups in it and B^1,3^ fragment at 215 Da indicating the presence of a pyrano group attached to ring B ring in addition to the presence of a hydroxyl group. The most important fragments detected for the fragmentation of isoflavones were due to loss of water, CO and CO_2_ in addition to RDA rearrangement fragments [19–21].

**3.1.1.5. Flavanes and isoflavanes**

One peak (**peak 21**) represented flavans glycoside class, it showed its quasi-molecular ion peak (M-H) at 405 Da along with its daughter peaks due to loss of pentose sugar, loss of water, CO and CO_2_ in addition to RDA rearrangement fragment A^1,3^ at 139 Da and a characteristic fragment at 295 Da that result from the loss of ring B [18]. On the other hand, 7 peaks (**peaks 59, 80, 110, 129, 130 and 133**) represented isoflavanes. All of them were identified as prenylated isoflavones except **peak 59**, the most important MS^2^ fragments were due to loss of ring B, CO, CO_2_ and water [18]. By referring to literature, **peaks 21, 59, 80, 110, 129, 130 and 133** were identified as tetrahydroxyflavan-O-pentoside, dihydroxy-methoxy isoflavane, gancaonin Y, glabridin, trihydroxy-diprenylisoflavan, hispaglabridin B, trihydroxy diprenyl isoflavane and dihydroxy-dimethoxy prenylisoflavane (Kovács et al. 2008;Montoro et al. 2011; Zhang et al. 2018;Simons 2011; Lim 2015; Öztürk et al. 2017).

**3.1.2 Triterpene saponins**

Triterpene saponins are considered as the key bioactive metabolites produced in licorice. The triterpene saponins of licorice are mostly of oleanane-type pentacyclic triterpene saponins and their chemical structure comprise a 30-carbon aglycone (sapogenin) along with multiple sugar connected units [23].

The course of our investigation on the chemical constituents of the different extracts of *Glycyrrhiza glabra* has led to the identification of 29 triterpenes, 28 of which are attached to sugars (triterpene saponins) [24].

The sugar moiety of triterpene saponins in Glycyrrhiza contains six basic sugar residues, including glucuronic acid residue (GluA), rhamnose residue (Rha), glucose residue (Glu), galacturonic acid residue (GalA), xylose residue (Xyl), and galactose residue (Gal) [24].

Most of the triterpenes were of the oleane-type. Only one saturated 27-carbon tetracyclic triterpene cholestane steroid (**peak 50**) was identified.

As of the standpoint of chemical structure, the aglycons of numerous oleanane-type pentacyclic glycosidic triterpene in glycyrrhiza genus have an a,b-unsaturated ketone element positioned at C-11, C-12, and C-13 as seen in **peaks 36, 45, 49, 53, 54, 55, 62, 65, 67, 76, 84, 118 and 126** [24].

Since the glycone of **peaks 32, 43, 44 and 49** is made up of –GlcA-GlcA-Rha group at the A ring, a major peak at m/z 497 [GlcA-GlcA-Rha–H]^−^ was detected in the four compounds [25]. Molecular formula of **peak** **32** was concluded to be C_48_H_74_O_21_. [M-H]^-^ was noticed at *m/z* 986. The negative ion at 351 indicated the presence of a saccharide chain with two units of glucuronic acid. The ion at *m/z* 489 is in accordance with [M-H–2GlcA–Rha]^+^. Based on the spectral analysis of **peak 32**, it was identified as yunganoside G1 [26]. **Compound 43** demonstrated a base peak at 453 (Aglycone-H-H_2_O)^-^ [27]. It spawned [M – H]^–^ at *m/z* 968 and had molecular formula C_48_H_72_O_20_ [28]. It generated fragment ion at *m/z* 833 [M – C_4_H_6_O_5_ – H]^–^ by losing a partial glycosyl segment, and then produced a fragment ion at *m/z* 645 [M – C_4_H_6_O_5_ – C_8_H_12_O_5_ – H] ^-^ with minimal abundance [6]. It was tentatively identified as yunganoside L1 or its isomer J1. Similarly, **peaks 44** and **49** was identified as Licorice saponin F3 and yunganoside K1 or its isomer, respectively [29].

Glycyrrhizic acid (β-glycyrrhizin) is the main licorice metabolite, one of the most representative saponins of *Glycyrrhiza*. Therefore **peak 67** was assigned to this component as it was the most intense peak in all studied samples. The quasi-molecular ion [M-H]^−^ (*m/z* 821) of glycyrrhizin was obvious. The *m/z* value of another fragment peak was 351. According to the structure of glycyrrhizin, the fragment came from the two glucuronic acids linked with its triterpene aglycon ([2 GlA - H]^-^) [30]. Its fragmentation pattern also showed ions at *m/z* 803 ([M-H_2_O-H]^-^), 645 ([M-glucouronic residue-H]^-^) [23][29][27]**. Compound 76** was assigned as α-glycyrrhizin, the conformational isomer of Glycyrrhizic acid isomer.

**Peaks 45, 46, 51, 54, 58, 61, 62, 64, 65, 84, 87 and 92** are glycyrrhizin derivatives with 2 glucuronic acid sugar moieties and are presented with a carboxyl group at C-30. The MS spectra of these compounds showed numerous ions, the most common were expressive of the successive losses of 2x176 Da (2xGlcA) in agreement with the existence of two glucuronic acids. One of the predominant fragment ions appears at *m/z* 351 [2GluA−H]^−^ corresponding to the saccharide chain. The base peak usually appears as [Aglycone+H-H_2_O]^+^ and is produced by abolition of H_2_O (18 Da) from the aglycone ion. A further typical loss of HCOOH (46 Da) was commonly observed due to a carboxyl group at C-30. In some cases, base peak ions process a series of cleavages and rearrangements in the A^-^ and B^-^ rings [31]. Formation of characteristic ion at *m/z* 317 Da, indicates the absence of substituent group at C-22 [31]. Successive or simultaneous losses of small molecules from the aglycone ion reproduced other daughter peaks at lower *m/z* values.

**Compound 45,** as an example**,** presented with molecular ions: [M-H]^-^ at *m/z* 895, corresponding to its molecular formula of C_44_H_64_O_19_. From [M-H]^-^, an ion, [M-H-GlcA]^-^ was produced at *m/z* 719 and the aglycone ion [M-H–2GlcA]^-^ at m/z 543. The base peak at *m/z* 525 [Aglycone-H-H_2_O]^-^ was produced by eradication of H_2_O (18 Da) from the aglycone ion. Consecutive or simultaneous losses of H_2_O, and/or AcOH and/or HCOOH from the m/z 525 spawned peaks at 507, 465, and 447. The ion at *m/z* 495 was generated from the base peak ion by a loss of 30 Da (CH_2_O), which was useful for differentiating some positional isomers involving the hydroxyl unit at C-24 or a different position. It was accordingly identified as Uralsaponin F [31].

The MS spectrum of **compound 84** showed a mass loss (2x176 Da) between the [M-H]^-^ and [Aglycone-H]^-^ ions, consistent a disaccharide composed of two glucuronic acids at C-3. The typical loss of 2x18 Da was observed in the MS spectrum, suggesting a hydroxyl group at C-22. Owing to the absence of the carboxyl group at C-30, the fragment ions in the MS spectra of **compound 84** was 15 Da less than those with a C-30 carboxyl[27]. It was thus identified as Uralsaponin C [30]. **Compound 92** was identified as yunganoside I_2_ [32].

**Compound 98** set forth a pseudomolecular ion at *m/z* 647 [M+H]^+^ in the MS spectrum, consistent with the molecular formula of C_36_H_54_O_10_. The spectrum revealed a major fragment ion at *m/z* 471 [M+H−176]^+^, proposing the presence of one glucuronic acid residue. The compound was identified as Yunganoside L [29]. The molecular formula of **compound 126** was calculated as C_36_H_52_O_10_ according to the MS spectrum (*m/z* 643 [M-H]^-^, it was accordingly identified as yunganoside E3.

Four peaks represented Oleanane type Triterpenes with lactone ring. Uralsaponin E (**peak 36**), licorice saponin F3 (**peak 44**), Yunganoside P (**peak 55**) and isoglaboride (**peak 118**), bear a 22(30)-lactone ring in place of the carboxyl substituent at C-30. In the spectra of this group of compounds, ring cleavages on the A- and B-rings as well as some neutral characteristic losses were like those of oleanane type triterpene saponin. The main difference was that the characteristic loss of 46 Da (HCOOH) was absent because of the presence of a 22(30)-lactone ring [31]. **Compounds 36 and 55** produced a base peak at 465 [Aglycone-H-H2O]^-^ , in addition, the characteristic loss of two sugar moieties (GlcA, 176 Da) spawned an ion at *m/z* 483 [M-H–2 GlcA]^-^ in both compounds. **Compound 55** revealed [M–H]^-^ ion at *m/z* 835 besides it also gave rise to an ion at *m/z* (437) due to loss of CH_2_O (30Da) corresponding to the presence of a C-24 hydroxyl group [24][30][33]. **Compound 55** was identified as Yunganoside P [33] and **Compound 36** was identified as Uralsaponin E. Mass spectrum of **compound 44** showed base peak ion (Aglycone-H-H_2_O)^-^ at *m/z* 436, in addition to anther characteristic fragment at *m/z* 418 (Aglycone-H-2H_2_O)^-^. The loss of 2 glucuronic acid moieties in addition to rhamnose lead us to identify **compound 44** as licorice saponin F3.

**3.1.3. Chalcones and chalcone glycosides**

Chalcones are phenolic plant shikimate pathway byproducts. They are composed of two rings; named A & B, linked via *α, β*- unsaturated carbonyl system with different substituents.[34]

Chalcones with hydroxyl group at position 2’, undergo Retro-Diels Alder (RDA) reaction represented by pathway a. This pathway results in very similar fragments to those resulting from their flavonone isomers. In case of chalcones lacking hydroxyl group at position 2’, cleavage occurs on either side of carbonyl group (pathway b) (Figure S2) [35].

Chalcones and chalcone glycosides were represented by 17 peaks. Chalcones were represented by 12 peaks (14, 47, 66, 71, 77, 89, 93, 96, 121, 122, 124 & 131) while peaks (23, 28, 35, 40 & 48) were assigned for chalcone glycosides.

Chalcones corresponding to peaks (47, 66, 77 & 122) undergo RDA reaction via pathway a resulting into two main daughter fragments; the one including ring A at 120 Da (M-H-C_8_H_8_) and the other with ring B at 104 Da (M-H-C_7_H_4_O_2_).

All chalcone glycosides fellow pathway a in their fragmentation owing to 2’ hydroxyl group in addition to daughter fragments resulting from loss of sugar units, additional prenyl or methyl groups.

Peak 28 was assigned for isoliquiritin apioside; the glycoside of peak of peak 66 identified as isoliquiritigenin. Peak 28 showed a fragment at 255 Da (M-H- sugar’’hexose and pentose units’’) in addition to the daughter fragments resulting from pathway a at 135 & 119 Da. Peak 61 showed directly the latter two fragments.

**3.1.4. Pterocarpans**

Pterocarpans are isoflavonoid derivatives of fused furan ring skeleton. They are composed of adjoining benzofuran and benzopyran rings.[36]

Pterocarpans were represented by four peaks (97, 106, 107 & 127) (Figure S3). Shinpterocarpin (compound 127) showed a quasi-molecular ion peak at m/z 321.3 Da. It follows typical fragmentation of pterocarpans resulting into two main daughter fragments corresponding to (C_7_H_6_O_2_ & C_14_H_12_O_2_) at m/z 122 & 212 respectively. In addition, a prominent fragment is resulted at m/z 146 corresponding to C_9_H_6_O_2_.

**3.1.5. Coumarins**

Coumarins are polyphenolic lactone compounds, composed of fused benzene and α-pyron rings.[37] Coumarins are represented by peaks (81&119) annotated as glabrocoumarin, and licocoumarin A respectively. They showed a sequential fragmentation (Figure S4).

Regarding licocoumarin A (compound 119), it showed typical fragmentation yielding a fragment of C_14_H_13_O_3_ (229 Da) that sequentially produce a prominent ion fragment of C_8_H_5_O at m/z 117 Da due to loss of CO_2_ (m/z 44). It also produced a fragment upon loss of both CO_2_ and HC. In addition to fragments resulting from isoprenyl group loss (M-H-C_5_H_9_) and (M-H- 2 C_5_H_9_) at m/z 336 and 267 respectively.

**3.1.6. Resorcinol derivatives**

Resorcinol is a phenolic meta-isomer of benzenediol, its derivatives are widely distributed among plants.[38]

Resorcinol derivatives were given by peaks (8, 9, 63 & 115). Peak 63 was identified as flavestin B (4-Prenyl-5-styrylresorcinol). This compound yielded quasi-molecular ion peak (M+H) at m/z 281and its subsequent fragments resulting from prenyl loss (M+H- C_5_H_9_) at m/z 212 and that produced after loss of both prenyl and styryl branches at m/z 111 (corresponding to resorcinol basic nucleus). Resorcinol nucleus then produced its characteristic fragments of C_6_H_11_ and C_6_H_9_ at m/z 83 and 81 respectively (Figure S5).

**References**

1. Aksay O, Selli S, Kelebek H. LC‐DAD‐ESI‐MS/MS-based assessment of the bioactive compounds in fresh and fermented caper (Capparis spinosa) buds and berries. Food Chem. 2021;337:127959.

2. Kadian N, Raju KSR, Rashid M, Malik MY, Taneja I, Wahajuddin M. Comparative assessment of bioanalytical method validation guidelines for pharmaceutical industry. J Pharm Biomed Anal. 2016;126:83–97.

3. Alberti-Dér Á. LC-ESI-MS/MS methods in profiling of flavonoid glycosides and phenolic acids in traditional medicinal plants: Sempervivum tectorum L. and Corylus avellana L. 2013.

4. Ghallab DS, Mohyeldin MM, Shawky E, Metwally AM, Ibrahim R said. Chemical profiling of Egyptian propolis and determination of its xanthine oxidase inhibitory properties using UPLC–MS/MS and chemometrics. LWT- Food Sci Technol. 2020;136:1–16.

5. Fabre N, Rustan I, Hoffmann E d., Quetin-Leclercq J. Determination of flavone, flavonol, and flavanone aglycones by negative ion liquid chromatography electrospray ion trap mass spectrometry. Am Soc Mass Spectrom. 2001;12:707–715.

6. Tsimogiannis D, Samiotaki M, Panayotou G, Oreopoulou V. Characterization of flavonoid subgroups and hydroxy substitution by HPLC-MS/MS. Molecules. 2007;12:593–606.

7. Ghallab DS, Mohyeldin MM, Shawky E, Metwally AM, Ibrahim RS. Chemical profiling of Egyptian propolis and determination of its xanthine oxidase inhibitory properties using UPLC – MS / MS and chemometrics. LWT. 2021;136:1–16.

8. Fukai T, Marumo A, Kaitou K, Kanda T, Terada S, Nomura T. Anti-Helicobacter pylori flavonoids from licorice extract. Life Sci. 2002;71:1449–63.

9. Suman A, Ali M, Alam P. New prenylated isoflavanones from the roots of Glycyrrhiza glabra. Chem Nat Compd. 2009;45:487.

10. Montoro P, Maldini M, Russo M, Postorino S, Piacente S, Pizza C. Metabolic profiling of roots of liquorice (Glycyrrhiza glabra) from different geographical areas by ESI/MS/MS and determination of major metabolites by LC-ESI/MS and LC-ESI/MS/MS. J Pharm Biomed Anal. 2011;54:535–44.

11. Zhang M, Deng Y, Wang C, Cai H-L, Wen J, Fang P-F, et al. An LC-MS/MS method for determination of bioactive components of liquorice and Semen Strychni in rat plasma: Application to a pharmacokinetics study. Drug Test Anal. 2018;10:262–71.

12. Lim TK. Glycyrrhiza glabra. Edible Med Non-Medicinal Plants. 2015;:354–457.

13. Xu T, Yang M, Li Y, Chen X, Wang Q, Deng W. An integrated exact mass spectrometric strategy for comprehensive and rapid characterization of phenolic compounds in licorice An integrated exact mass spectrometric strategy for comprehensive and rapid characterization of phenolic compounds in licorice. Rapid Commun Mass Spectrom. 2013;:2297–2309.

14. Krasteva I, Nikolov S. Flavonoids in Astragalus corniculatus. Quim Nova. 2008;31:59–60.

15. Jing W, Yan R, Wang Y. A practical strategy for chemical profiling of herbal medicines using ultra-high performance liquid chromatography coupled with hybrid triple quadrupole-linear ion trap mass spectrometry: a case study of Mori Cortex. Anal Methods. 2015;7:443–57.

16. Kovács A, Vasas A, Hohmann J. Natural phenanthrenes and their biological activity. Phytochemistry. 2008;69:1084–110.

17. Wei S, Wu W, Ji Z. New antifungal pyranoisoflavone from ficus tikoua bur. Int J Mol Sci. 2012;13:7375–82.

18. Simons R. Prenylated isoflavonoids from soya and licorice: Analysis, induction and in vitro estrogenicity. Wageningen University.; 2011.

19. Nakata R, Yoshinaga N, Teraishi M, Okumoto Y, Huffaker A, Schmelz EA, et al. A fragmentation study of isoflavones by IT-TOF-MS using biosynthesized isotopes. Biosci Biotechnol Biochem. 2018;82:1309–15.

20. Raju KSR, Kadian N, Taneja I, Wahajuddin M. Phytochemical analysis of isoflavonoids using liquid chromatography coupled with tandem mass spectrometry. Phytochem Rev. 2015;14:469–98.

21. Kang J, Hick LA, Price WE. A fragmentation study of isoflavones in negative electrospray ionization by MSn ion trap mass spectrometry and triple quadrupole mass spectrometry. Rapid Commun Mass Spectrom. 2007;21:857–68.

22. Öztürk M, Altay V, Hakeem KR, Akçiçek E. Pharmacological activities and phytochemical constituents: liquorice. Springer, cam; 2017.

23. Montero L, Ibáñez E, Russo M, di Sanzo R, Rastrelli L, Piccinelli AL, et al. Metabolite profiling of licorice (Glycyrrhiza glabra) from different locations using comprehensive two-dimensional liquid chromatography coupled to diode array and tandem mass spectrometry detection. Anal Chim Acta. 2016;913:145–59.

24. Li F, Liu B, Li T, Wu Q, Xu Z, Gu Y, et al. Review of constituents and biological activities of triterpene saponins from Glycyrrhizae Radix et Rhizoma and its solubilization characteristics. Molecules. 2020;25:3904.

25. Wang S, Chen P, Xu Y, Li X, Fan X. Characterization of the chemical constituents in Da-Huang-Gan-Cao-Tang by liquid chromatography coupled with quadrupole time-of-flight tandem mass spectrometry and liquid chromatography coupled with ion trap mass spectrometry. J Sep Sci. 2014;37:1748–61.

26. Shou Q, Jiao P, Hong M, Jia Q, Prakash I, Hong S, et al. Triterpenoid saponins from the roots of Glycyrrhiza Glabra. Nat Prod Commun. 2019;14:1934578X1901400106.

27. Ohtani K, Kasai R, Yang CR, Yamasaki K, Zhou J, Tanaka O. Oleanane glycosides from roots of Glycyrrhiza yunnanensis. Phytochemistry. 1994;36:139–45.

28. Chen L-L, Chen C-H, Zhang X-X, Wang Y, Wang S-F. Identification of constituents in Gui-Zhi-Jia-Ge-Gen-Tang by LC-IT-MS combined with LC-Q-TOF-MS and elucidation of their metabolic networks in rat plasma after oral administration. Chin J Nat Med. 2019;17:803–21.

29. Ji S, Wang Q, Qiao X, Guo H, Yang Y, Bo T, et al. New triterpene saponins from the roots of Glycyrrhiza yunnanensis and their rapid screening by LC/MS/MS. J Pharm Biomed Anal. 2014;90:15–26.

30. Xie J, Zhang Y, Wang W, Hou J. Identification and simultaneous determination of glycyrrhizin, formononetin, glycyrrhetinic acid, liquiritin, isoliquiritigenin, and licochalcone A in licorice by LC-MS/MS. Acta Chromatogr. 2014;26:507–16.

31. Zheng Y-F, Qi L-W, Zhou J-L, Li P. Structural characterization and identification of oleanane-type triterpene saponins in Glycyrrhiza uralensis Fischer by rapid-resolution liquid chromatography coupled with time-of-flight mass spectrometry. Rapid Commun Mass Spectrom. 2010;24:3261–70.

32. Ma X, Guo X, Song Y, Qiao L, Wang W, Zhao M, et al. An integrated strategy for global qualitative and quantitative profiling of Traditional Chinese Medicine formulas: Baoyuan decoction as a case. Sci Rep. 2016;6:38379.

33. Xu T, Pi Z, Liu S, Song F, Liu Z. Chemical profiling combined with “Omics” technologies (CP-Omics): a strategy to understand the compatibility mechanisms and simplify herb formulas in Traditional Chinese Medicines. Phytochem Anal. 2017;28:381–91.

34. Tekale S, Mashele S, Pooe O, Thore S, Kendrekar P, Pawar R. Biological role of chalcones in medicinal chemistry. In: Vector-Borne Diseases. Rijeka: IntechOpen; 2020.

35. Xu T, Yang M, Li Y, Chen X, Wang Q, Deng W, et al. An integrated exact mass spectrometric strategy for comprehensive and rapid characterization of phenolic compounds in licorice. Rapid Commun Mass Spectrom. 2013;27:2297–309.

36. Militão GCG, Pinheiro SM, Dantas INF, Pessoa C, de Moraes MO, Costa-Lotufo LC V, et al. Bioassay-guided fractionation of pterocarpans from roots of Harpalyce brasiliana Benth. Bioorg Med Chem. 2007;15:6687–91.

37. Lončar M, Jakovljević M, Šubarić D, Pavlić M, Buzjak Služek V, Cindrić I, et al. Coumarins in food and methods of their determination. Foods. 2020;9:645.

38. Christensen LP. Polyphenols and polyphenol-derived compounds from plants and contact dermatitis. In: Watson RR, Preedy VR, Zibadi S, editors. Polyphenols: Prevention and Treatment of Human Disease. Second Edi. Academic Press; 2018. p. 349–84.

39. Zhang J, Xu X-J, Xu W, Huang J, Zhu D, Qiu X-H. Rapid characterization and identification of flavonoids in Radix Astragali by Ultra-High-Pressure Liquid Chromatography coupled with Linear Ion Trap-Orbitrap Mass Spectrometry. J Chromatogr Sci. 2014;53:945–52.

40. Ren Z, Nie B, Liu T, Yuan F, Feng F, Zhang Y, et al. Simultaneous determination of coumarin and its derivatives in Tobacco products by Liquid Chromatography-Tandem Mass Spectrometry. Molecules. 2016;21.

41. Mahendra Kumar T, Alice B, Dahryn T, Gopa N, Parthasarathi P, Snehasis J. Evaluation of the isotopic abundance ratio in biofield energy treated resorcinol using Gas Chromatography-Mass Spectrometry Technique. Pharm Anal Acta. 2016;7.


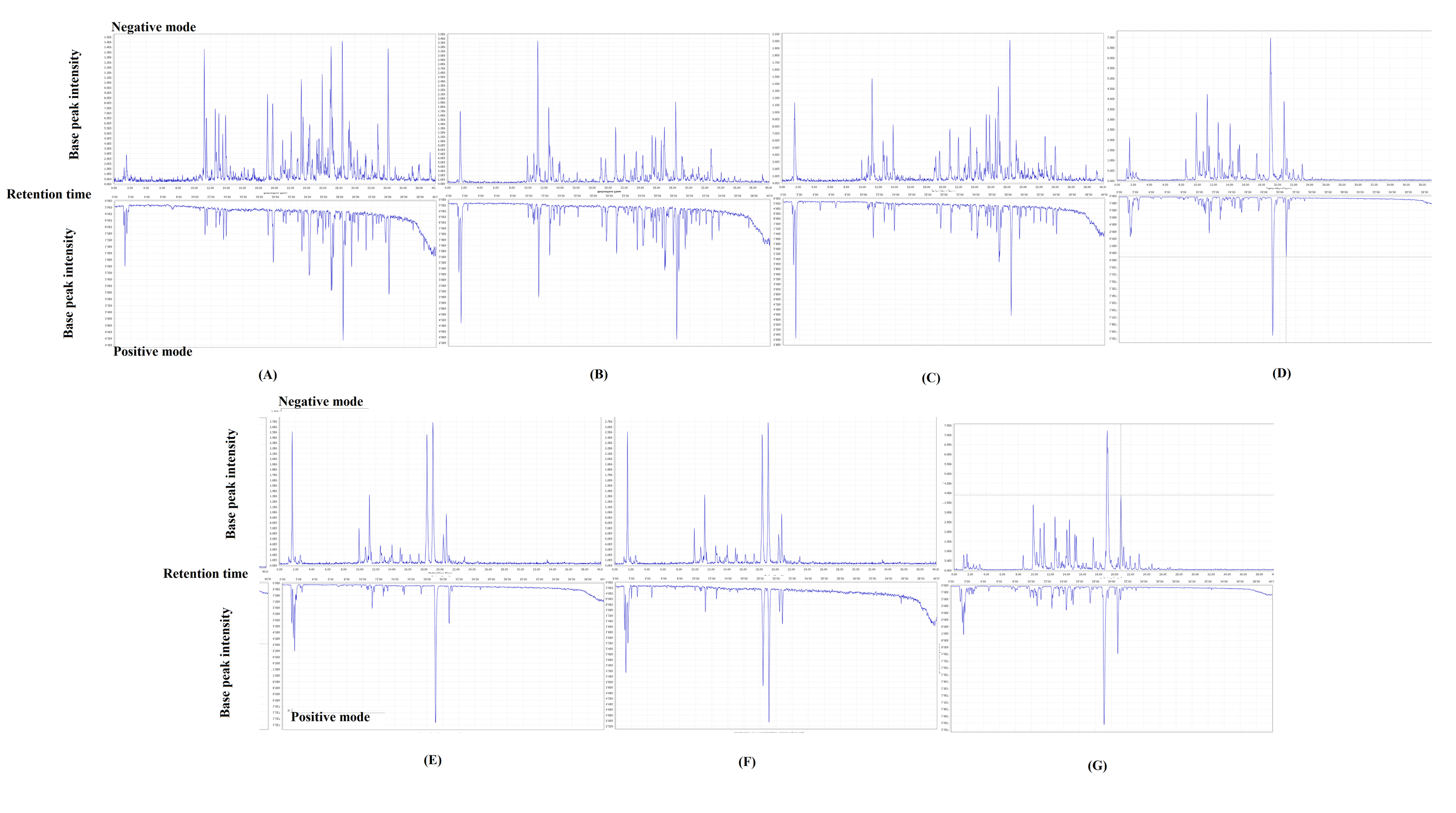


Figure S1: Base peak chromatograms of (A) ethanol extract of roasted sample, (B) ethanol extract of raw sample, (C) ethanol extract of honey-roasted sample, (D) aqueous extract of fermented sample, (E) aqueous extract of roasted sample, (F) aqueous extract of honey roasted sample, (G)aqueous extract of raw sample.


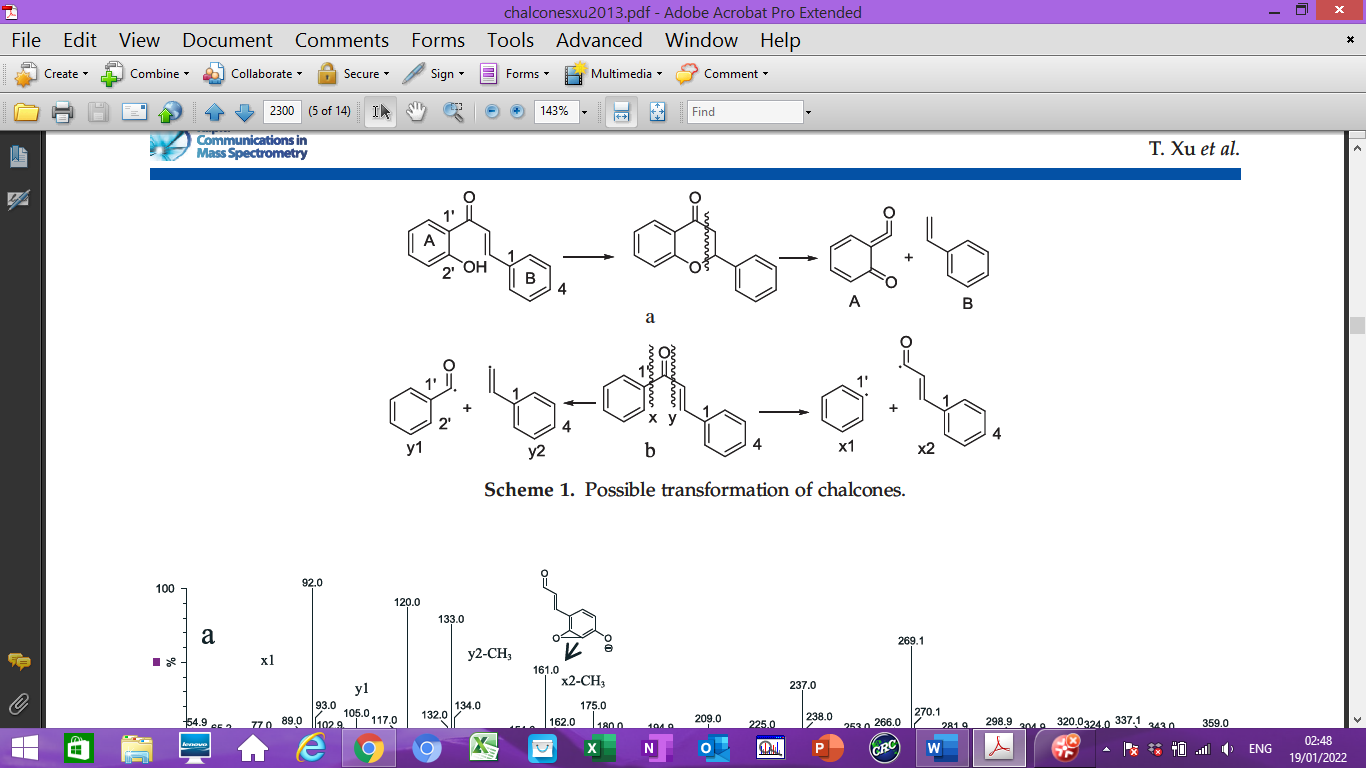


Figure S2: Possible fragmentation of chalcones [35]


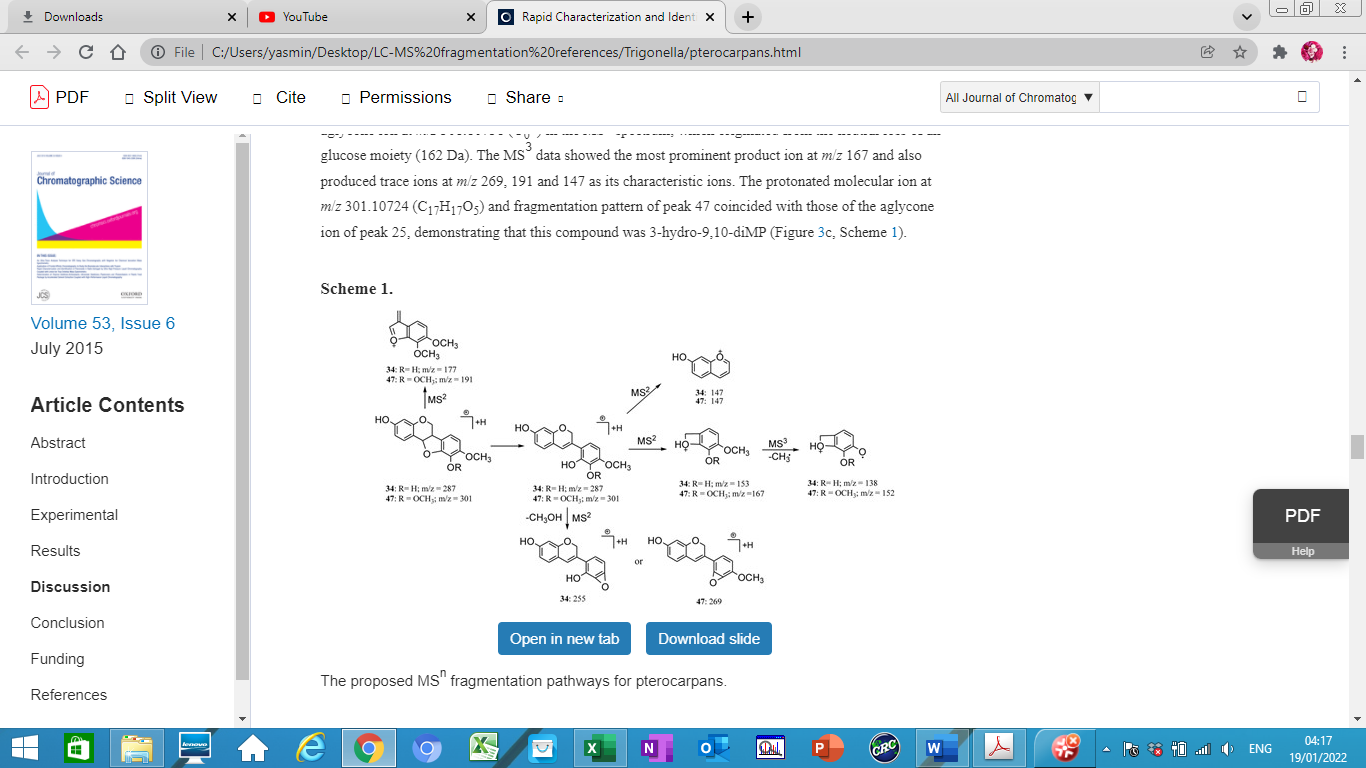


Figure S3: Possible fragmentation of pterocarpans [39]


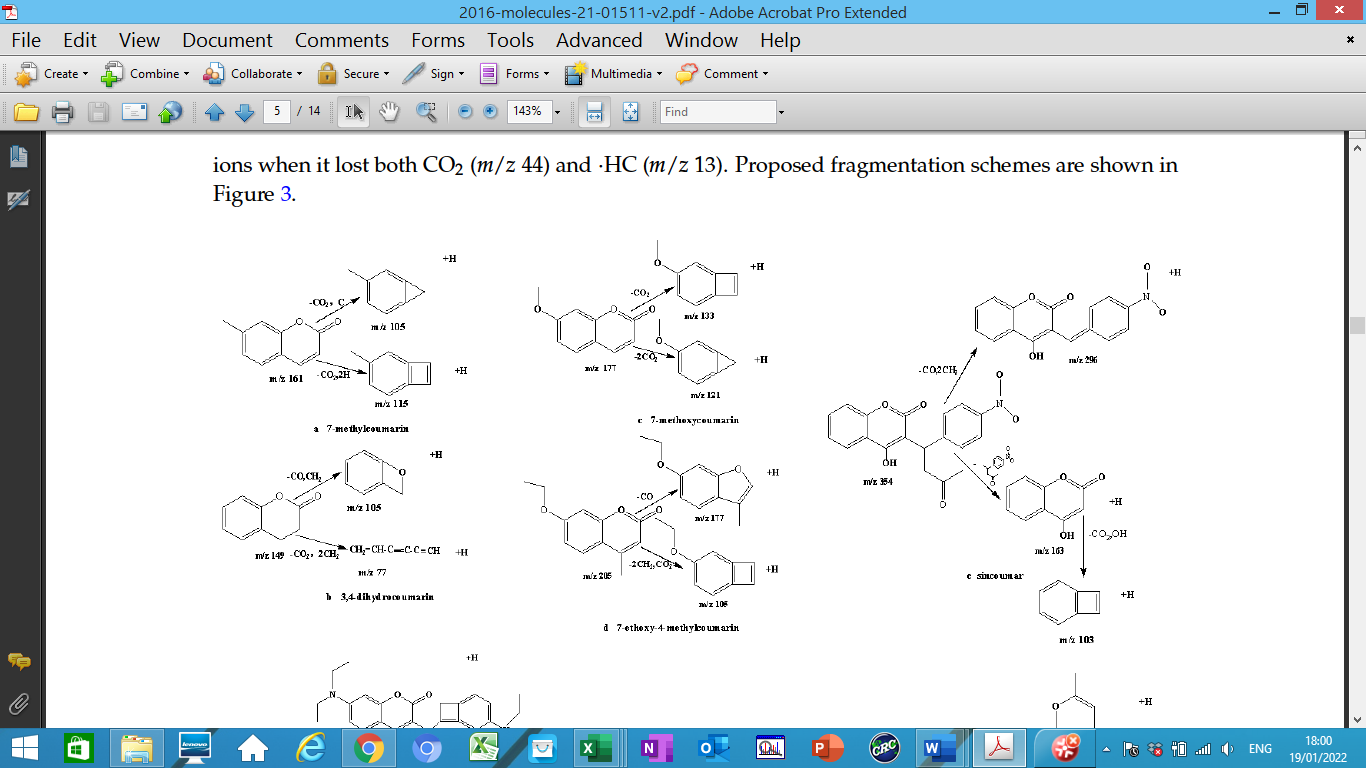


Figure S4: Possible fragmentation of coumarins [40]


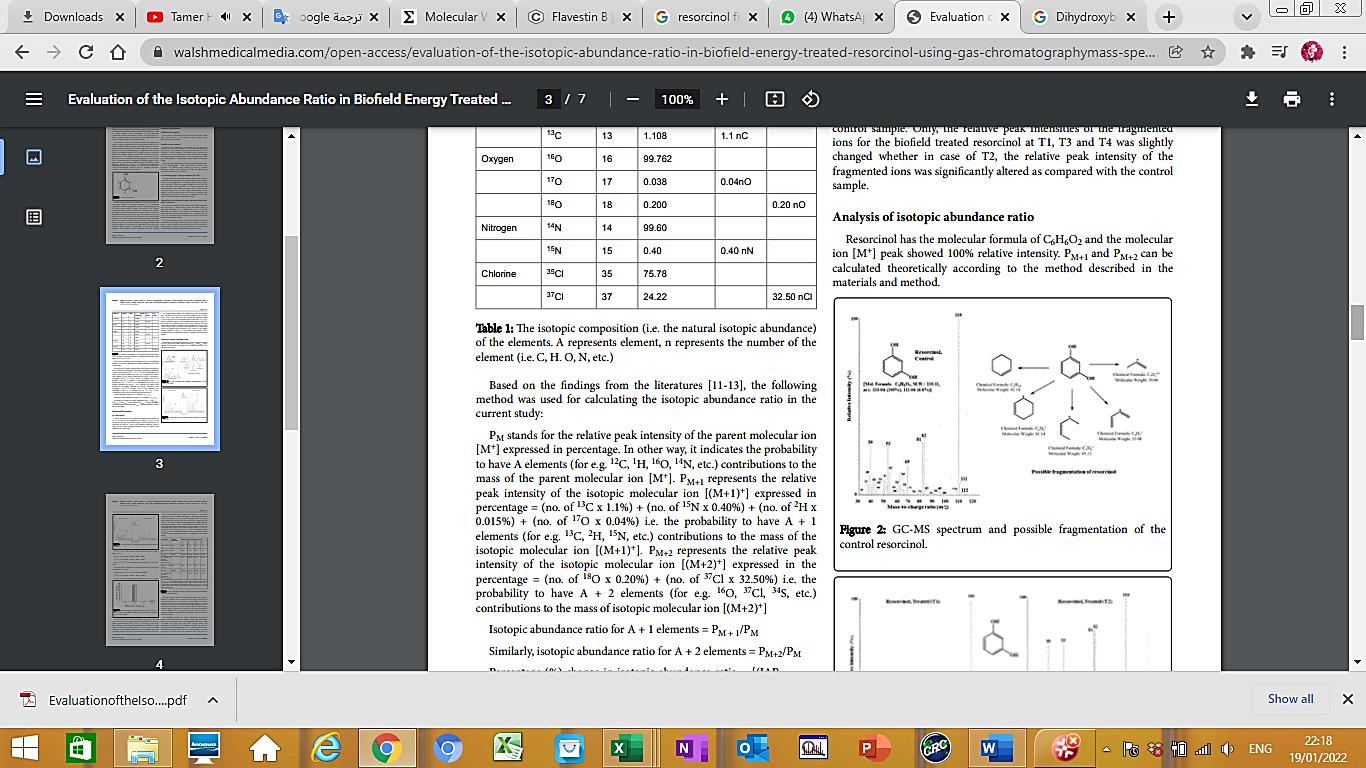


Figure S5: Possible fragments of resorcinol [41]
